# Targeting the Notch1‐YY1‐ICAM1 Signaling Axis Enhances the Efficacy of Immunotherapy in HCC by Activating CD8^+^ T‐Cell‐Driven Cancer Cell Pyroptosis

**DOI:** 10.1002/advs.202512845

**Published:** 2025-11-21

**Authors:** Ke Zhu, Fa‐Peng Zhang, Chao Qin, Zhi‐Xiao Song, Cai‐Ni Yang, Shu‐Sheng Lin, Xian‐Huan Yu, Wen‐Rui Wu, Chao‐Qun Liu, Chao Liu, Lei‐Bo Xu

**Affiliations:** ^1^ Guangzhou Key Laboratory of Precise Diagnosis and Treatment of Biliary Tract Cancer Department of Biliary‐Pancreatic Surgery Sun Yat‐sen Memorial Hospital, Sun Yat‐sen University Guangzhou 510120 China; ^2^ Guangdong Provincial Key Laboratory of Malignant Tumor Epigenetics and Gene Regulation Guangdong‐Hong Kong Joint Laboratory for RNA Medicine Sun Yat‐sen Memorial Hospital Sun Yat‐sen University Guangzhou 510120 China; ^3^ Department of Pathology Guangdong Provincial People's Hospital Guangdong Academy of Medical Sciences Guangzhou 510080 China; ^4^ Scientific Research Center The Seventh Affiliated Hospital Sun Yat‐sen University Shenzhen 518000 China

**Keywords:** hepatocellular carcinomas, Notch1, pyroptosis, tumor‐specific CD8^+^ T cells, YY1

## Abstract

Immunotherapy has shown a modest clinical benefit in advanced hepatocellular carcinoma (HCC), probably due to tumor immunosuppressive functions. Although Notch1 signaling has been implicated in tumor immune escape, its underlying mechanism is unclear. Here, Notch1 signaling is established as a determinant of immunotherapy efficacy in HCC. Studies showed that high Notch1 expression correlates with poor progression‐free survival and worse immunotherapeutic response in recurrent HCC patients, while Notch1 overexpression promotes cancer cell escape by inhibiting CD8^+^ T cell activation. Mechanistically, Notch1 overexpression upregulates the expression of transcriptional factor YY1 (Yin‐Yang 1), which in turn represses ICAM1 expression to prohibit CD8^+^ T cell‐derived granzyme‐driven cancer cell pyroptosis and cytotoxicity. Finally, co‐administration of a PD‐L1 antibody with PEI‐siYY1 represses HCC tumor growth without causing severe adverse effects, as observed with the Notch1 inhibitor DAPT. The results establish that targeting Notch1‐YY1‐ICAM1 signaling axis may enhance immunotherapy efficacy by activating CD8^+^ T cell‐driven cancer cell pyroptosis, providing a safe and effective treatment strategy for HCC patients.

## Graphical abstract

In hepatocellular carcinoma (HCC), aberrantly activated Notch1 signaling induces its target gene YY1, which impairs immunotherapy efficacy by suppressing tumor cell‐ICAM1‐driven T cell activation and the mediated pyroptosis of T cells against tumor cells. This establishes the Notch1‐YY1‐ICAM1 signaling axis as a safe and effective therapeutic target for a combination immunotherapy strategy.

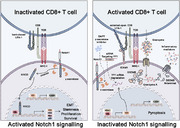

## 1 Introduction

Hepatocellular carcinoma (HCC) is the fourth leading cause of cancer‐related mortality worldwide, with a 5‐year survival rate of less than 18%.^[^ ^1^ ^]^ Current first‐line therapies, including surgical resection, liver transplantation, or radiofrequency ablation, are only suitable for patients with early‐stage HCC, whereas ≈60% of HCC patients are diagnosed at a late stage.^[^ ^2^ ^]^ Although treatment with the multityrosine kinase inhibitor sorafenib shows modest clinical benefit in advanced HCC patients, the majority of patients become resistant to this therapy during the treatment period.^[^ ^3^ ^]^ To address this problem, immune checkpoint inhibitors (ICIs), such as the PD‐1/PD‐L1 monoclonal antibodies nivolumab and atezolizumab, have been approved by the FDA for use in patients with a history of sorafenib treatment.^[^ ^4^, ^5^, ^6^ ^]^ Nevertheless, the objective response rate of these HCC patients after ICI treatment is only 15%.^[^ ^7^ ^]^ Therefore, there is an unmet clinical need to improve the clinical performance of immune checkpoint inhibitors in advanced HCC patients.

Previous studies from our group and others have shown that the Notch1 signaling pathway plays a prominent role in HCC development and progression.^[^ ^8^, ^9^ ^]^ Interestingly, accumulating evidence suggests that the administration of a Notch1 inhibitor can reduce the infiltration of immunosuppressive cells, such as myeloid‐derived suppressor cells (MDSCs), regulatory T cells (Tregs), and tumor‐associated macrophages (TAMs), into tumors while increasing the infiltration of cytotoxic T cells (CTLs) in several tumors in vivo,^[^ ^10^, ^11^ ^]^ indicating that pharmacological modulation of the Notch1 signaling pathway may improve immunotherapy efficacy. Although targeting the Notch1 signaling pathway with either Notch1 inhibitors, γ‐secretase inhibitors (GSIs) or Notch1‐specific monoclonal antibodies has shown satisfactory clinical benefits in the early trials of triple‐negative breast cancer,^[^ ^12^ ^]^ glioma,^[^ ^13^ ^]^ and lymphoma,^[^ ^14^ ^]^ these drugs cause certain off‐target effects and toxicity in patients, especially dose‐dependent diarrhea caused by the intestinal toxicity of GSIs.^[^ ^15^ ^]^ Present solutions for adverse reactions include intermittent administration or a combination of glucocorticoids. However, intermittent administration does not ensure sufficient antitumor effects, and the combination of glucocorticoids may lead to more serious adverse effects. Thus, targeting the downstream effector of Notch1 signaling, which is responsible for its immunosuppressive function, may be a better strategy for enhancing immunotherapy treatment efficacy in HCC.

Cytotoxic lymphocyte‐mediated cancer cell death includes apoptosis, necrosis, pyroptosis, etc.^[^ ^16^ ^]^ Pyroptosis is a form of gasdermin‐mediated programmed cell death that frequently occurs after infection with intracellular pathogens.^[^ ^17^ ^]^ Zhou et al. reported that cytotoxic lymphocytes can secrete granzyme A (GZMA) to induce gasdermin B (GSDMB)‐driven cancer cell pyroptosis, whereas activating granzyme A (GZMA)‐driven GSDMB cleavage prohibits tumor growth in mice.^[^ ^18^ ^]^ Previous studies have shown that ICAM1 is critical for T lymphocyte activation.^[^ ^19^ ^]^ However, it is unknown whether HCC cells upregulate Notch1 expression to downregulate ICAM1 expression to prevent T lymphocyte activation and subsequent granzyme‐driven cancer cell pyroptosis.

Here, we investigated the role of the Notch1 signaling pathway in modulating immunotherapy efficacy. Clinically, high expression of Notch1 correlated with poor progression‐free survival in HCC patients after receiving adjuvant PD‐1/PD‐L1 antibody treatment, whereas depletion of the Notch1 intracellular domain (N1ICD) (i.e., the active form of Notch1) strongly enhanced anti‐PD‐L1 efficacy against orthotopic HCC tumor growth in vivo. Mechanistically, aberrant expression of N1ICD in cancer cells transcriptionally upregulated the expression of the transcriptional repressor Yin‐Yang 1 (YY1) to reduce ICAM1 expression by binding to its promoter, which in turn prohibited CD8^+^ T‐cell cytotoxicity and subsequent immunotherapy resistance. Importantly, depletion of cancer cell‐N1ICD or YY1 stimulated CD8^+^ T‐cell activation to increase granzyme‐driven cancer cell pyroptosis, whereas silencing GSDMB expression in N1ICD‐depleted HCC cells prohibited CD8^+^ T‐cell cytotoxicity against cancer cells. Finally, the administration of the PEI‐YY1‐targeting siRNA complex enhanced the efficacy of the PD‐L1 antibody against orthotopic liver growth without causing adverse side effects. Overall, these findings demonstrated that targeting the expression of YY1, rather than its upstream regulator Notch1, could be a safe and effective way to increase immunotherapy efficacy in HCC patients.

## 2 Results

### 2.1 High Notch1 Expression Is Correlated with Poor Progression‐Free Survival and worse Poor Immunotherapeutic Response in HCC Patients after Receiving Adjuvant PD‐1/PD‐L1 Antibody Treatment

**Figure 1:**
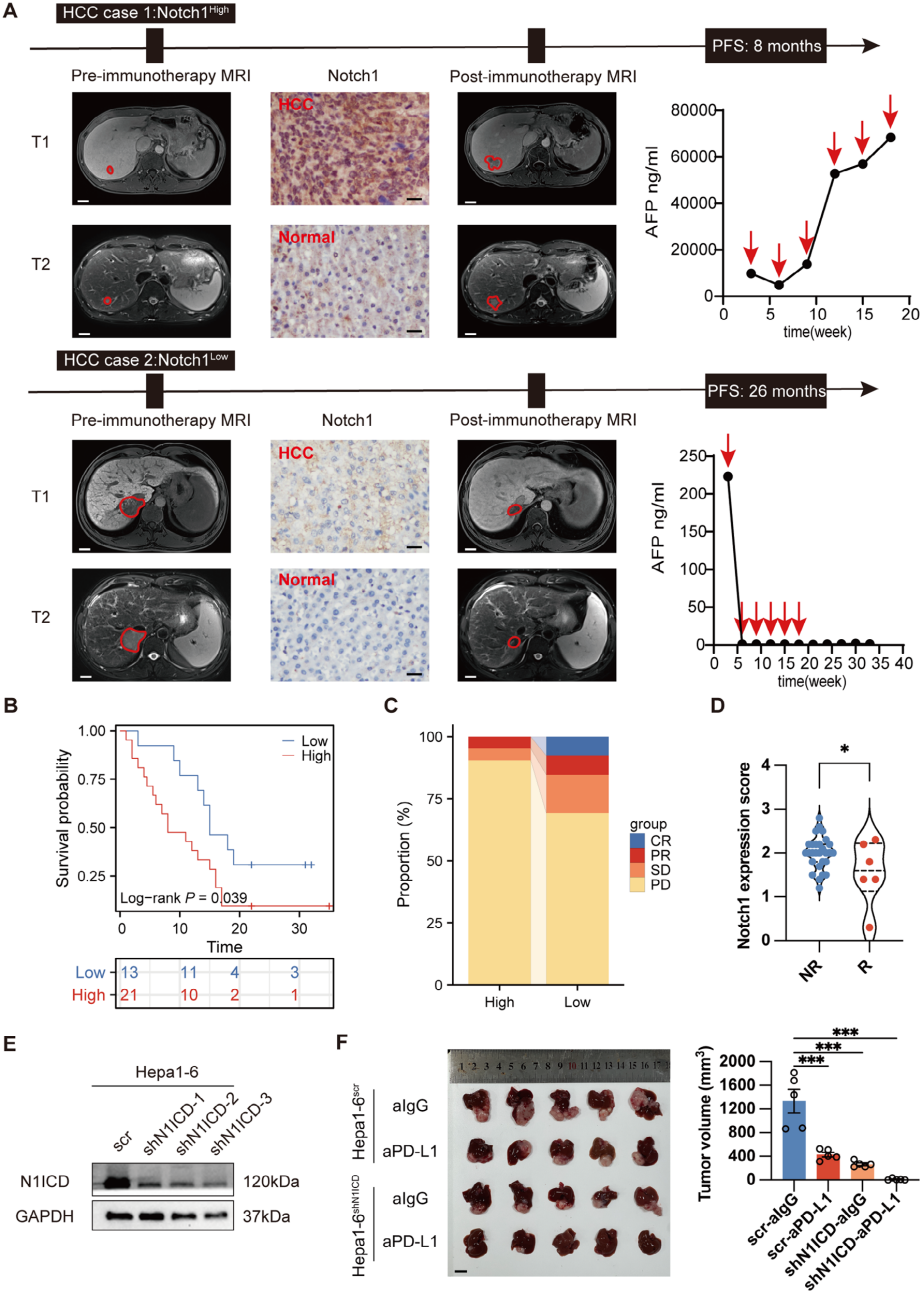
High Notch1 expression indicates poor therapeutic efficacy of immune checkpoint inhibitors and poor outcomes in HCC patients. A) Therapeutic response to anti‐PD‐1/PD‐L1 monoclonal antibodies in two representative HCC patients with high or low N1ICD expression. Representative CT images, immunohistochemical staining of N1ICD and PD‐L1, serum AFP levels, and PFS data are shown. The tumor border is marked by red lines in the MR images. The red arrow indicates the timing of anti‐PD‐1/PD‐L1 treatment in HCC patients. B) High N1ICD expression correlated with poor progression‐free survival in HCC patients after receiving adjuvant anti‐PD‐L1/PD‐1 treatment (*n* = 34 HCC patients; cohort 1). C) Therapeutic response to immunotherapy in HCC patients with low or high N1ICD expression according to the mRECIST guidelines according to the CT/MRI results. CR, complete response; PR, partial response; PD, progressive disease; SD, stable disease. D) Immunotherapeutic response in HCC patients with high or low N1ICD (*n* = 34 HCC patients; cohort 1). Each sample on the violin plots represents individual patient data (NR = nontonder, R = responder). E) Western blot analysis confirmed the knockdown of N1ICD in Hepa‐1‐6 cells. F) C57BL/6 mice were orthotopically injected with Hepa‐1‐6^shN1ICD^ or Hepa‐1‐6^scr^. The liver tumor‐bearing mice were intraperitoneally injected with 5 mg kg^−1^ PD‐L1 antibody or IgG control antibody on day 7 and treated twice a week for up to 2 weeks (*n* = 5 mice per group). A representative gross tumor image from each group is shown. The bar chart shows the final tumor volume of each group. The means ± SEMs are given. **p < 0.01, ***p < 0.001, ****p < 0.0001. B) Log‐rank test. D) Student's *t*‐test. The scale bars in (A) represent 2 cm (white), A) 100 µm (black), and F) 1 cm.

Previous work from our laboratory and others has shown that the expression of the Notch1 intracellular domain (N1ICD) is correlated with poor prognosis and cancer progression in HCC patients, whereas Qiu et al. reported that Notch1 inhibition may enhance immunotherapy efficacy in melanoma in vivo.^[^ ^20^ ^]^ We therefore sought to explore the role of Notch1 expression in determining the cancer immunotherapeutic response of HCC patients. A cohort of 34 HCC patients who received complete surgical resection and adjuvant anti‐PD‐1/PD‐L1 antibody treatment was recruited. Tumor recurrence status was monitored by performing computerized tomography (CT) scanning/magnetic resonance imaging (MRI) and measuring the serum alpha‐fetoprotein (AFP) level in these HCC patients. The expression of Notch1 and the tumor recurrence status of two representative anti‐PD‐L1 antibody‐treated HCC patients were assessed. Our results revealed that high Notch1 expression was correlated with poor PFS and rapid tumor recurrence in HCC patients after receiving anti‐PD‐1/PD‐L1 antibody treatment, whereas low Notch1 expression was associated with increased PFS and reduced tumor relapse (**Figure** **1**A–D; Table S1, Supporting Information), suggesting that the level of Notch1 expression could predict the immunotherapeutic response in HCC patients. To examine the functional role of Notch1 in modulating anti‐PD‐L1 efficacy, we first established stable N1ICD‐knockdown (Hepa1‐6^shN1ICD^) and N1ICD‐overexpressing (Hepa1‐6^N1ICD^) mouse HCC Hepa1‐6 cell lines (Figure 1E; Figure S1A,B, Supporting Information), which were then orthotopically injected into the livers of C57BL/6 mice. Once the tumors became palpable, the tumor‐bearing mice were then treated with either an anti‐PD‐L1 monoclonal antibody (aPD‐L1) or an anti‐IgG control antibody (IgG). Our results showed that depleting N1ICD enhanced the anti‐PD‐L1 antibody efficacy against Hepa‐1‐6 tumor growth compared with that in the anti‐PD‐L1 antibody‐treated scramble control group (Figure 1F). Further immunohistochemistry studies revealed that the number of CD8^+^ T cells was greater in Hepa1‐6^shN1ICD^ orthotopic liver tumors than in the scramble control group (Figure S1C,D, Supporting Information), which is consistent with previous observations. Overall, our results demonstrated for the first time that high expression of Notch1 is associated with poor immunotherapeutic response in HCC patients, while it is also correlated with anti‐PD‐L1 antibody efficacy in vivo.

### 2.2 N1ICD Expression in Cancer Cells Determines Human Cytotoxic T Lymphocyte Cytotoxicity In Vitro and In Vivo

**Figure 2:**
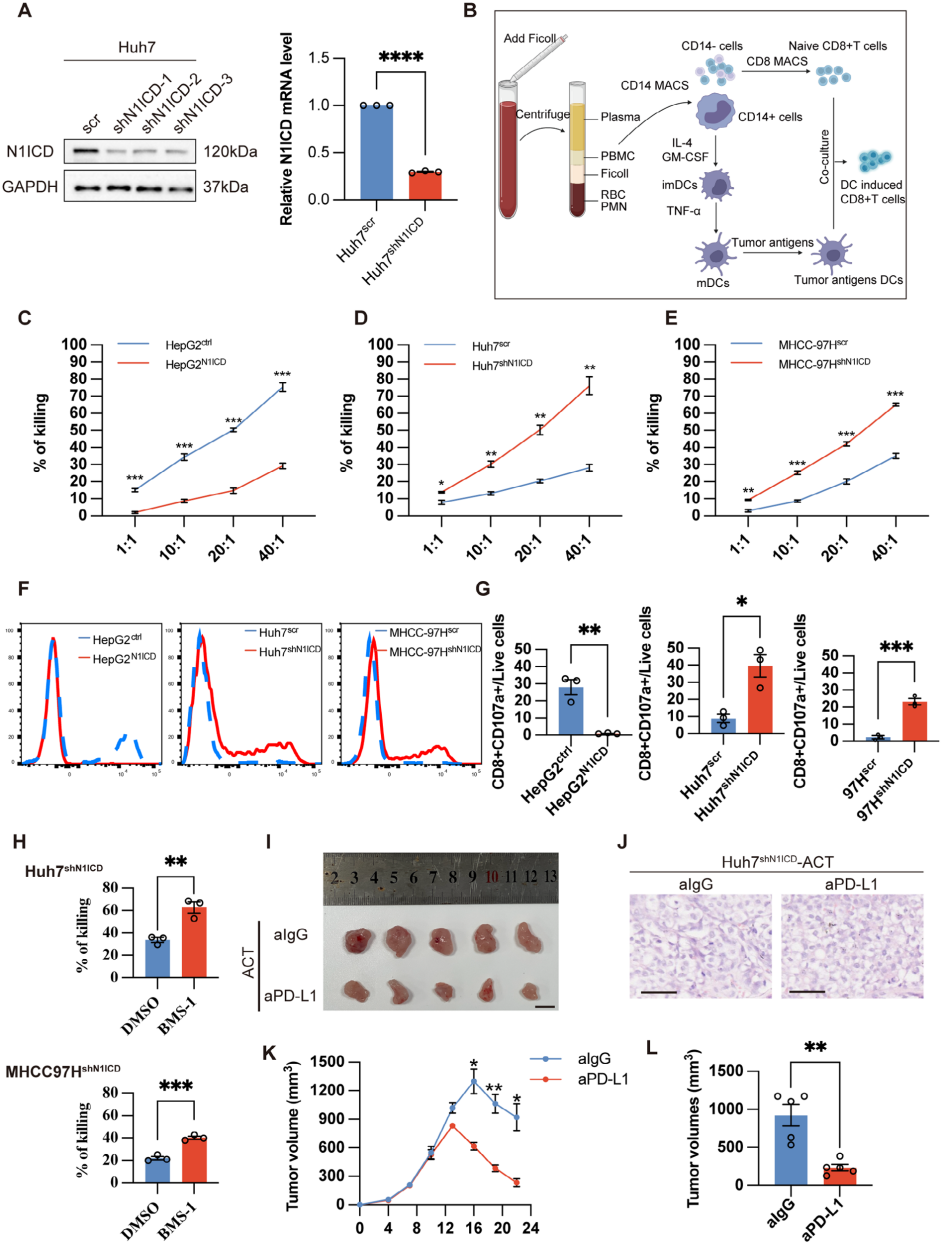
N1ICD expression in cancer cells regulates human cytotoxic T lymphocyte‐mediated killing in vitro and in vivo. A) Both Western blot and RT‒PCR confirmed the knockdown of N1ICD in Huh7 cells. B) Preparation process of cancer cell lysate‐pulsed dendritic cells primed with CD8^+^ T cells (created with BioRender.com). C–E) LDH release assay of HepG2^N1ICD^/HepG2^ctrl^ cells, Huh7^shN1ICD^/Huh7^scr^ cells or MHCC‐97H^shN1ICD^/MHCC‐97H^scr^ cells after co‐culture with tumor‐specific CD8^+^ T cells at different E/T ratios as indicated (*n * = 3 independent experiments). F) Flow cytometry analysis of CD107a expression on tumor‐specific CD8^+^ T cells after co‐culture with HepG2^N1ICD^/HepG2^ctrl^ cells, Huh7^shN1ICD^/Huh7^scr^ cells or MHCC‐97H^shN1ICD^/MHCC‐97H^scr^ cells (E/T ratio: 10:1) as indicated. G) Bar chart showing the ratio of CD8^+^CD107a^+^ T cells in each group. H) LDH release assays of Huh7^shN1ICD^/MHCC‐97H^shN1ICD^ cells after co‐culture with tumor‐specific CD8^+^ T cells (E/T ratio: 10:1) in the presence of the DMSO solvent control or BMS‐1 (10 µm). I) NOD/SCID mice were subcutaneously injected with 3 × 10^6^ Huh7^shN1ICD^ cells, which were treated with either an anti‐PD‐L1 (aPD‐L1) or anti‐IgG (IgG) antibody together with adoptive cell transfer (ACT: tumor‐specific CD8^+^ T cells). A representative gross tumor image from each group is shown. J) Representative images of H&E‐stained sections from each group are shown. K) Tumor growth curves of Huh7^shN1ICD^ cells after treatment with tumor‐specific CD8^+^ T cells in the presence of aPD‐L1 or IgG (*n* = 5 mice per group). L) Bar chart showing the final tumor volume in each treatment group (*n* = 3 mice per group). The means ± SEMs are given. *p < 0.05, **p < 0.01, ***p < 0.001, ****p < 0.0001. A,C–E,G,H,K,L) Student's *t*‐test. The scale bars in (I) represent 1 cm, and those in (J) represent 50 µm.

To validate the immunosuppressive role of N1ICD, we first established stable N1ICD‐knockdown models in human HCC cell lines (Huh7 and MHCC97H) and an N1ICD‐overexpressing model in HepG2 cells (**Figure** **2**A; Figure S2A–C, Supporting Information). Using a co‐culture system with activated human CD8^+^ T cells (effector, E) and HCC cells (target, T) at various E:T ratios (1:1, 10:1, 20:1, 40:1), we observed that N1ICD overexpression significantly attenuated T‐cell‐mediated cancer cell killing, whereas N1ICD knockdown reversed this immunosuppressive phenotype (Figure S2D, Supporting Information). To determine whether this effect was antigen‐specific, tumor‐specific CD8^+^ T cells were generated by priming peripheral blood mononuclear cell (PBMC)‐derived CD8^+^ T cells with HCC lysate‐pulsed dendritic cells (DCs) for 14 days (Figure 2B). Our results revealed that tumor‐specific CD8^+^ T cells were less effective at killing stable N1ICD‐expressing cells than were empty vector‐transfected cells (Figure 2C), while they significantly eliminated more N1ICD‐depleted HCC cells than were scramble‐transfected cells (Figure 2D,E), suggesting that tumor‐specific CD8^+^ T cells should be used in the rest of this study. Since CD107a is known as a marker of CD8^+^ T‐cell cytotoxic activity following stimulation (i.e., degranulation),^[^ ^21^, ^22^ ^]^ we next performed flow cytometry analysis of CD107a expression in tumor‐specific CD8^+^ T cells after coculturing with either stable N1ICD‐expressing HepG2 cells (HepG2^N1ICD^)/control empty vector‐transfected cells (HepG2^ctrl^) or N1ICD‐depleted Huh7 (Huh7^shN1ICD^)/MHCC‐97H (MHCC97H^shN1ICD^) cells/scramble‐transfected cells (Huh7^scr^/MHCC97Hcr), which indicated that coculturing tumor‐specific CD8^+^ T cells with stable N1ICD‐expressing HCC cells reduced CD107a expression compared with that in the group co‐cultured with empty vector‐transfected HCC cells, whereas CD107a expression was upregulated in tumor‐specific CD8^+^ T cells after co‐culture with N1ICD‐depleted HCC cells compared with that in the group co‐cultured with scramble‐transfected cells (Figure 2F,G). Since HCC cell lines express PD‐L1, we next tested whether treatment with BMS‐1 (an inhibitor of PD‐1 and PD‐L1 protein‒protein interactions in human cells) could further affect tumor‐specific CD8^+^ T‐cell cytotoxicity against N1ICD‐depleted HCC cells. Our results indicated that the administration of BMS‐1 enhanced tumor‐specific CD8^+^ T‐cell‐mediated cytotoxicity in N1ICD‐depleted HCC cells compared with that in scramble‐transfected cells (Figure 2H). We next sought to confirm our observations in vivo by performing adoptive cell transfer (ACT) therapy in the presence or absence of aPD‐L1. NOD/SCID mice were subcutaneously injected with Huh7^shN1ICD^ cells, while the tumor‐bearing mice were treated with tumor‐specific CD8^+^ T cells (i.e., adoptive cell transfer therapy, ACT) together with IgG or aPD‐L1 (Figure S2E, Supporting Information). Our results indicated that combined treatment with ACT and aPD‐L1 most significantly repressed HCC tumor growth compared with the groups treated with ACT plus aIgG (Figure 2I‒L). Overall, our results indicate that cancer cell‐specific N1ICD expression may determine tumor‐specific CD8^+^ T‐cell cytotoxicity and immunotherapy efficacy in vitro and in vivo via an unknown mechanism.

### 2.3 N1ICD Expression in Cancer Cells Prohibits CD8^+^ T‐Cell‐Derived Granzyme‐Mediated GSDMB‐Driven Cancer Cell Pyroptosis

**Figure 3:**
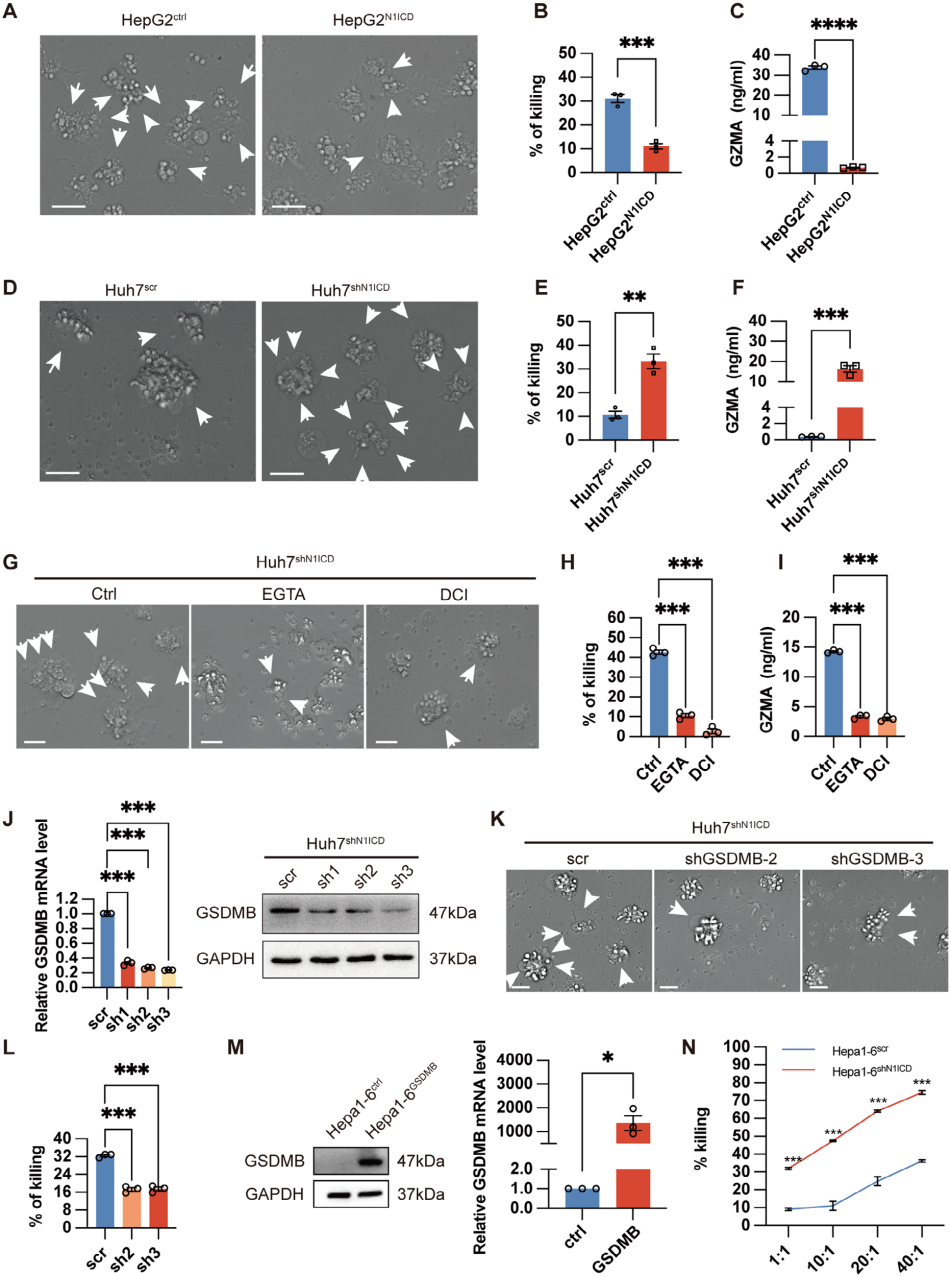
N1ICD expression in cancer cells prohibits tumor‐specific CD8^+^ T‐cell‐secreted granzyme‐mediated GSDMB‐driven cancer cell pyroptosis. A) Representative high‐throughput automated confocal images of HepG2^N1ICD^/HepG2^ctrl^ cells after co‐culture with tumor‐specific CD8^+^ T cells (E:T ratio 10:1) are shown. The white arrows indicate pyroptotic cells. B) LDH release assay of HepG2^N1ICD^/HepG2^ctrl^ cells after co‐culture with tumor‐specific CD8^+^ T cells (*n* = 3 independent experiments). C) ELISAs of GZMA secretion in the culture medium of tumor‐specific CD8^+^ T cells after co‐culture with HepG2^N1ICD^/HepG2^ctrl^ cells. D) Representative high‐throughput automated confocal images of Huh7^shN1ICD^/Huh7^scr^ cells after co‐culture with tumor‐specific CD8^+^ T cells (E:T ratio 10:1) are shown. E) LDH release assay of Huh7^shN1ICD^/Huh7^scr^ cells after co‐culture with tumor‐specific CD8^+^ T cells (*n* = 3 independent experiments). F) ELISAs of GZMA secretion in the culture medium of tumor‐specific CD8^+^ T cells after co‐culture with Huh7^shN1ICD^/Huh7^scr^ cells. G) Representative high‐throughput automated confocal images of cancer cells after co‐culture with tumor‐specific CD8^+^ T cells in the presence of the granzyme inhibitor EGTA or CD8^+^ T cells pretreated with the pangranzyme inhibitor DCI are shown. H) LDH release of cancer cells after co‐culture with either tumor‐specific CD8^+^ T cells in the presence of the granzyme inhibitor EGTA or tumor‐specific CD8^+^ T cells pretreated with the pangranzyme inhibitor DCI as indicated. I) ELISAs of GZMA secretion in the culture medium of tumor‐specific CD8^+^ T cells after co‐culture with Huh7^shN1ICD^/Huh^scr^ cells. J) Both Western blot and RT‒qPCR confirmed the knockdown of GSDMB expression in Huh7^shN1ICD^ cells. K) Representative high‐throughput automated confocal images of GSDMB‐silenced Huh7^shN1ICD^ cells/scramble‐transfected cells after co‐culture with tumor‐specific T cells are shown. L) LDH assays of GSDMB‐silenced Huh7^shN1ICD^ cells/scramble‐transfected Huh7shN1ICD cells. (*n* = 3 independent experiments). M) Both Western blot and RT‒qPCR confirmed the overexpression of GSDMB in Hepa1‐6 cells. N) LDH release assay of Hepa1‐6^shN1ICD^ cells/Hepa1‐6^scr^ cells after co‐culture with tumor‐specific CD8^+^ T cells at different E/T ratios as indicated (*n* = 3 independent experiments). The means ± SEMs are given. **p < 0.01, ***p < 0.001, ****p < 0.0001. B,C,E,F,H–J,L–N) Student's *t*‐test. The scale bars in (A,D,G,K) represent 50 µm.

Recent studies have indicated that the cytotoxicity of activated CD8^+^ T cells is partly attributed to secreted granzyme A (GZMA)‐mediated gasdermin B (GSDMB)‐driven cancer cell pyroptosis,^[^ ^18^ ^]^ while the induction of cancer cell pyroptosis has been shown to promote checkpoint inhibitor efficacy in cancer treatment, probably through the release of inflammatory factors such as IL‐18 and IL‐1β.^[^ ^23^ ^]^ However, it is still unknown whether cancer cell‐N1ICD expression can regulate immunotherapy efficacy via pyroptosis in HCC. We next examined whether coculturing HCC cells with tumor‐specific CD8^+^ T cells could induce pyroptosis. By employing a high‐throughput automated confocal imaging technique, our results indicated that tumor‐specific CD8^+^ T cells were less effective at inducing pyroptosis in HepG2^N1ICD^ cells than were empty vector‐transfected cells, while CD8^+^ T cells induced more pyroptosis in Huh7^shN1ICD^ cells than did scramble‐transfected cells. This observation was further confirmed by LDH release assays (i.e., an indicator of cell pyroptosis) and GZMA ELISA, which revealed that the percentage of LDH release (i.e., the percentage of killing) and the GZMA concentration were significantly lower in stable N1ICD‐expressing HepG2 cells after treatment with tumor‐specific CD8^+^ T cells than in empty vector‐transfected cells (**Figure** **3**A–C), whereas LDH release was greater in Huh7^shN1ICD^ cells after treatment with tumor‐specific CD8^+^ T cells than in scramble‐transfected cells (Figure 3D–F), suggesting that cancer cell‐N1ICD expression reflects tumor‐specific CD8^+^ T‐cell‐mediated cancer cell pyroptosis. To confirm the role of tumor‐specific CD8^+^ T‐cell‐derived granzymes in inducing cancer cell pyroptosis, we co‐cultured HCC cells with either tumor‐specific CD8^+^ T cells in the presence/absence of the granzyme inhibitor EGTA or tumor‐specific CD8^+^ T cells pretreated with the pangranzyme inhibitor DCI, which were then subjected to high‐throughput automated confocal imaging. Our results indicated that the number of pyroptotic cells was significantly lower in HCC cells after co‐culture with either tumor‐specific CD8^+^ T cells in the presence of EGTA or tumor‐specific CD8^+^ T cells pretreated with the pangranzyme inhibitor DCI than in untreated cells, while this observation was further confirmed by an LDH release assay (Figure 3G–I), suggesting that tumor‐specific CD8^+^ T cells induce cancer cell pyroptosis by secreting GZMA.

To identify the key executioner of pyroptosis in this co‐culture system, we overexpressed gasdermin family members (GSDMA, GSDMB, GSDMC, GSDMD, and GSDME) in HepG2 cells and performed cytotoxicity assays with CD8^+^ T cells. LDH release assays revealed that, compared with empty vector control cells, GSDMB‐overexpressing cells presented significantly increased LDH levels (Figure S3A, Supporting Information). These data strongly suggest that GSDMB is the predominant mediator of pyroptosis in this tumor‐T‐cell interaction model. To further examine the role of cancer cell GSDMB expression in CD8^+^ T‐cell‐derived GZMA‐driven pyroptosis, we stably knocked down GSDMB expression in Huh7^shN1ICD^ cells (Figure 3J), which were then co‐cultured with tumor‐specific CD8^+^ T cells. Our results indicated that knocking down GSDMB expression in Huh7^shN1ICD^ cells reduced tumor‐specific CD8^+^ T‐cell‐induced cancer cell pyroptosis and LDH release compared with those in Huh7^scr^ cells (Figure 3K,L). We also validated these findings in mouse HCC cells. First, following the methodology of Shao et al.,^[^ ^18^ ^]^ we overexpressed GSDMB in Hepa1‐6 cells (Figure 3M) and co‐cultured these cells with tumor‐specific CD8+ T cells. LDH release assays revealed that N1ICD knockdown significantly reduced the killing activity of CD8+ T cells (Figure 3N). Overall, our work demonstrated that cancer cell‐specific N1ICD expression regulated tumor‐specific CD8^+^ T‐cell‐derived granzyme‐mediated GSDMB‐driven cancer cell pyroptosis and its mediated cytotoxicity.

### 2.4 Cancer Cell‐N1ICD Regulates ICAM1 Expression to Modulate Tumor‐Specific T‐Cell Cytotoxicity and Mediate Cancer Cell Pyroptosis

**Figure 4:**
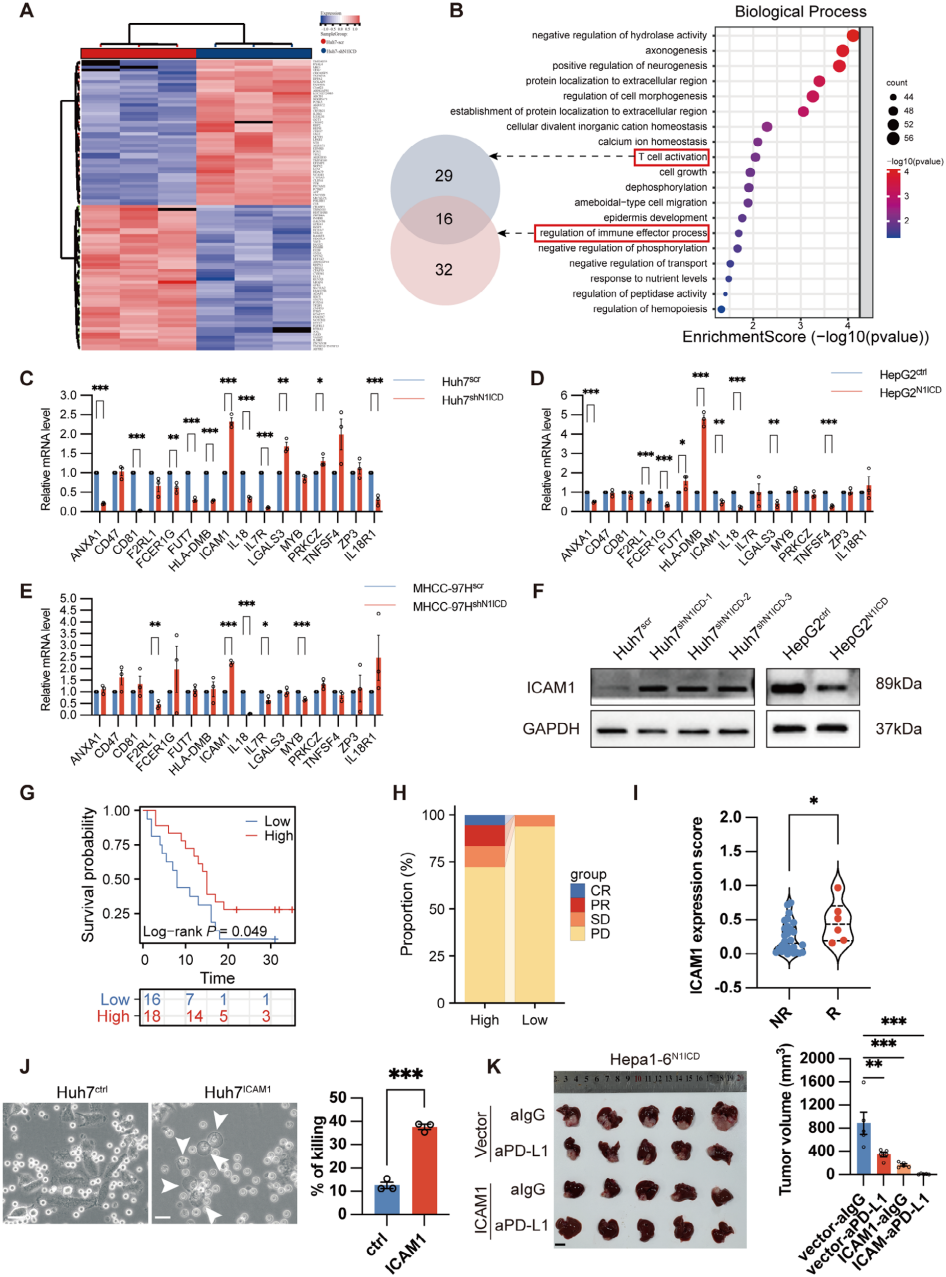
N1ICD regulates ICAM expression in cancer cells to determine the immunotherapeutic response in HCC. A) Heatmap showing the RNA sequencing results of N1ICD‐depleted Huh7 cells compared with scramble‐transfected cells. B) GO enrichment analysis revealed a significant difference in several biological processes between Huh7^shN1ICD^ cells and Huh7^scr^ cells. C) Venn diagram analysis of the GO biological processes revealed that the expression of *IL18R1, CD47, LGALS3, ANXA1, TNFSF4, ZP3, ICAM1, IL7R, CD81, FUT7, MYB, IL18, PRKCZ, FCER1G, HLA‐DMB, and F2RL1* was altered in Huh7^shN1ICD^ cells compared with Huh7^scr cells^. D) RT‒qPCR analysis of altered gene expression in HepG2^N1ICD^/HepG2^ctrl^ cells, Huh7^shN1ICD^/Huh7^scr^ cells, and MHCC‐97H^shN1ICD^/MHCC‐97H^scr^ cells (*n* = 3 independent experiments). F) Western blot analysis of ICAM1 expression in HepG2^N1ICD^/HepG2^ctrl^ cells or Huh7^shN1ICD^/Huh7^scr^ cells. G) Low ICAM1 expression correlated with poor progression‐free survival in HCC patients who received anti‐PD‐1/PD‐L1 antibody treatment (*n* = 34 HCC patients; cohort 1). H) Therapeutic response to immunotherapy in HCC patients with low or high ICAM1 expression according to the mRECIST guidelines. I) Immunotherapeutic response in HCC patients with high or low ICAM1 (*n* = 34 HCC patients, cohort 1). Each sample on the violin plots represents individual patient data. J) Representative high‐throughput automated confocal images and LDH release assay of ICAM1‐overexpressing Huh7 (Huh7^ICAM1^)/control empty vector‐transfected (Huh7^ctrl^) cells after co‐culture with tumor‐specific CD8^+^ T cells. K) At 7 days after orthotopic Hepa‐1‐6^N1ICD^ cell injection, the tumor‐bearing mice were treated with the PEI‐ICAM1 complex (2 mg kg^−1^, i.v.) every three days for up to 7 days together with IgG or aPD‐L1 (5 mg kg^−1^, i.p.) every 3 days for a total of 3 times. A representative gross tumor image from each treatment group is shown. The bar chart shows the final tumor volume in each group (*n* = 5 mice per group). The means ± SEMs are given. *p < 0.05, **p < 0.01, ***p < 0.001, ****p < 0.0001. C–E,I–K) Student's t test. (G) Log‐rank test. Scale bars in (L) represent 50 µm, (K) 1 cm.

To further explore the molecular mechanism by which cancer cell‐specific N1ICD expression inhibits tumor‐specific CD8^+^ T‐cell cytotoxicity, we performed RNA‐sequencing analysis with Huh7^shN1ICD^ and Huh7^scr^ cells and identified 746 upregulated and 772 downregulated genes in Huh7^shN1ICD^ cells compared with those in Huh7^scr^ cells (**Figure** **4**A). Further Gene Ontology (GO) enrichment analysis revealed several significantly enriched biological processes, including T‐cell activation and regulation of immune effector processes, etc., in Huh7^shN1ICD^ cells compared with Huh7^scr^ cells (Figure 4B; Figure S4A,B, Supporting Information), whereas KEGG pathway enrichment analysis revealed that pathways involved in protein digestion and absorption, cytokine‒cytokine receptor interactions, linoleic acid metabolism, etc., were particularly enriched in Huh7^shN1ICD^ cells (Figure S4C, Supporting Information). Venn diagram analysis revealed that the expression of genes involved in T‐cell activation and the regulation of immune effector processes, such as *IL18R1*, *CD47*, *LGALS3*, *ANXA1*, *TNFSF4*, *ZP3*, *ICAM1*, *IL7R*, *CD81*, *FUT7*, *MYB*, *IL18*, *PRKCZ*, *FCER1G*, *HLA‐DMB*, and *F2RL1*, was differentially expressed in Huh7^shN1ICD^ cells compared with Huh7^scr^ cells (Figure 4B). To confirm our observations, we performed RT‒qPCR analysis of the expression of these altered genes in Huh7^shN1ICD^/MHCC‐97H^shN1ICD^ cells as well as in HepG2^N1ICD^ cells, which revealed that, compared with that in empty vector/scramble‐transfected cells, the expression of ICAM1 was consistently altered in N1ICD‐modified HCC cells (Figure 4C‒E). Since Notch1 has been shown to modulate ICAM1 expression in endothelial cells via an unknown mechanism, ICAM1 is an important mediator of lymphocyte activity. We therefore focused on the downstream role of ICAM1 in Notch1‐mediated immunosuppression by examining the protein expression of ICAM1 in our HCC cell lines and found that N1ICD‐depleted cells presented greater ICAM1 expression than scramble‐transfected cells did, whereas ICAM1 expression was downregulated in HepG2^N1ICD^ cells compared with HepG2^ctrl^ cells (Figure 4F). To confirm the clinical significance of our findings, we performed an IHC study with our HCC cohorts, which revealed that low expression of ICAM1 was correlated with poor PFS, increased tumor recurrence and a worse therapeutic response in HCC patients after receiving anti‐PD‐1/PD‐L1 antibody treatment (Figure 4G–I). We stably overexpressed ICAM1 in Huh7 cells and observed a significant increase in pyroptosis after coculturing ICAM1‐overexpressing cells with tumor‐specific CD8^+^ T cells (Figure S4D, Supporting Information; Figure 4J). Next, we designed and labeled ICAM1‐targeting siRNA (siICAM1)/nonsilencing control siRNA (siNSC) with 2′OMe modification, as well as ICAM1 plasmids and empty control vector plasmids, which were then mixed with an in vivo/jet polyethylenimine (PEI) regent (i.e., PEI‐siICAM1, PEI‐siNSC, PEI‐Vector, or PEI‐ICAM1 complex) and used for animal experiments. Orthotopic liver tumor models demonstrated that ICAM1 overexpression reversed N1ICD‐mediated immunosuppression, whereas ICAM1 knockdown abrogated the immunotherapeutic benefits of N1ICD depletion, collectively indicating that ICAM1 is a key enhancer of immunotherapy efficacy. (Figure 4K; Figure S4E, Supporting Information). These results suggest that N1ICD may regulate ICAM1 expression to modulate CD8^+^ T‐cell activity.

**Figure 5:**
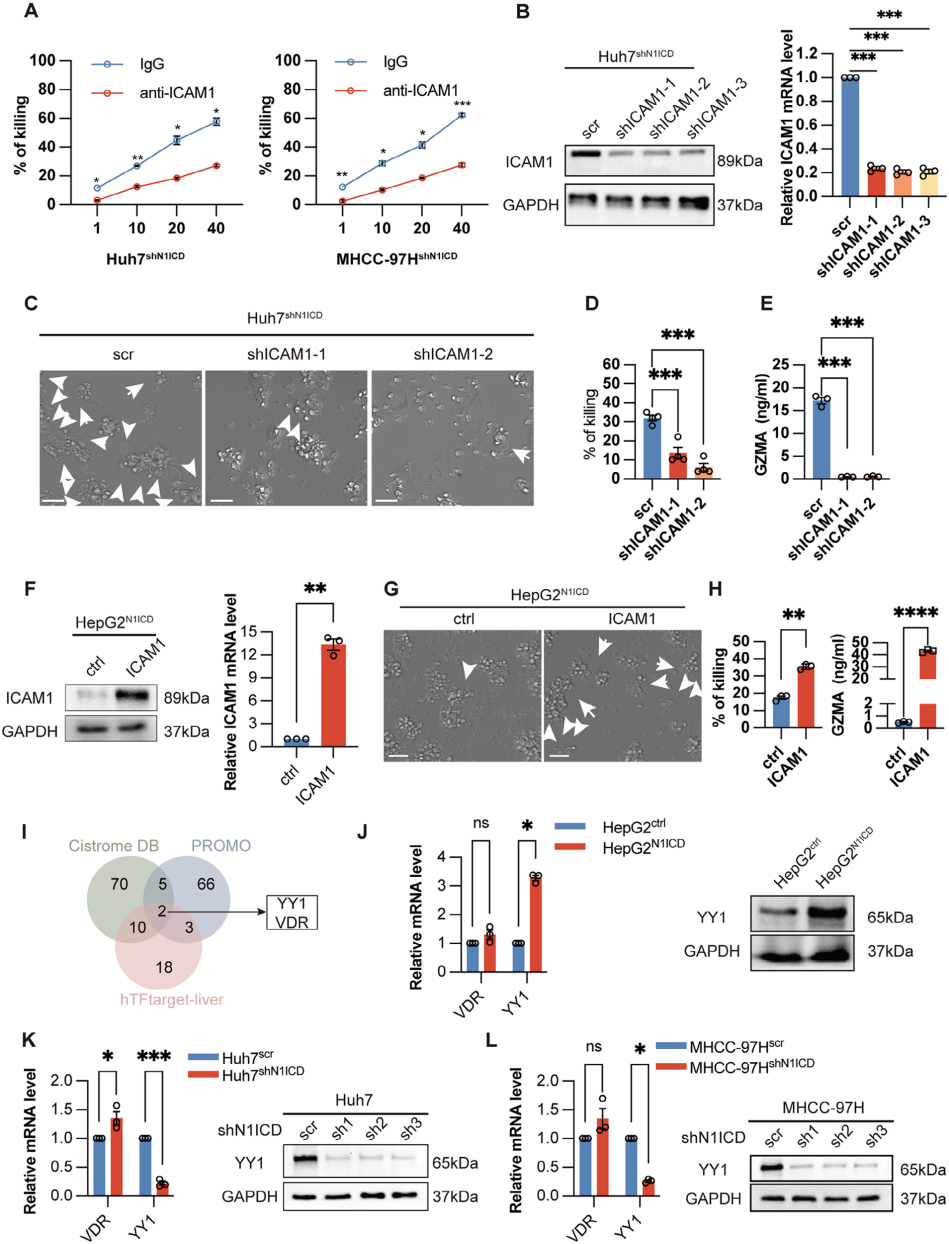
Cancer cell‐N1ICD downregulates ICAM1 expression to prohibit tumor‐specific CD8^+^ T‐cell cytotoxicity and mediate cancer cell pyroptosis. A) LDH release assay of Huh7^shN1ICD^/MHCC‐97H^shN1ICD^ cells after treatment with tumor‐specific CD8^+^ T cells at different E/T ratios in the presence of the IgG control or ICAM‐1 neutralizing antibody. B) Both Western blot and RT‒qPCR confirmed the knockdown of ICAM1 expression in Huh7^shN1ICD^ cells. C) Representative high‐throughput automated confocal images of ICAM1‐silenced Huh7^shN1ICD^ cells/scramble‐transfected cells after co‐culture with tumor‐specific CD8^+^ T cells are shown. D) LDH release assay of ICAM1‐silenced Huh7^shN1ICD^ cells/scramble‐transfected cells after co‐culture with tumor‐specific CD8^+^ T cells. E) ELISAs of GZMA expression in the culture medium of ICAM1‐silenced Huh7^shN1ICD^ cells/scramble‐transfected cells after co‐culture with tumor‐specific CD8^+^ T cells. F) Both Western blot and RT‒qPCR confirmed the expression of ICAM1 in HepG2^N1ICD^ cells. G) Representative high‐throughput automated confocal images of stable ICAM1‐expressing HepG2^N1ICD^ cells and control cells after treatment with tumor‐specific CD8^+^ T cells. H) LDH release assay and GZMA ELISA of stable ICAM1‐expressing HepG2^N1ICD^ cells and control cells after treatment with tumor‐specific CD8^+^ T cells (*n* = 3 independent experiments). I) Venn diagram analysis showing the transcription factor‐binding site prediction of the ICAM1 promoter via three different online databases (i.e., CistromeDB, PROMO, and hTFtarget (liver)). J–L) Both RT‒PCR and Western blot analysis revealed that YY1 expression was upregulated in HepG2^N1ICD^ cells compared with that in HepG2^ctrl^ cells, whereas YY1 expression was downregulated in Huh7^shN1ICD^/MHCC‐97H^shN1ICD^ cells compared with that in scramble‐transfected cells. The means ± SEMs are given. *p < 0.05, **p < 0.01, ***p < 0.001, ****p < 0.0001. A,B,D–F,H,J–L) Student's *t*‐test. The scale bars in (C,G) represent 50 µm.

To verify the downstream effector role of ICAM1 in Notch1‐mediated tumor‐specific T‐cell activity, we co‐cultured N1ICD‐depleted HCC cells with tumor‐specific CD8^+^ T cells at different E/T ratios in the presence of IgG control or ICAM1 neutralizing antibodies. Strikingly, our cytotoxicity assays indicated that treatment with an ICAM1 neutralizing antibody prohibited the cytotoxicity of tumor‐specific CD8^+^ T cells to N1ICD‐depleted cells compared with that in the group treated with an IgG control antibody (**Figure** **5**A). Furthermore, we first stably knocked down ICAM1 in Huh7^shN1ICD^ cells (Figure 5B) and co‐cultured these cells with tumor‐specific CD8^+^ T cells (E/T ratio: 10:1) for pyroptosis and cytotoxicity assays. Strikingly, high‐throughput automated confocal imaging revealed that silencing ICAM1 in Huh7^shN1ICD^ cells reduced tumor‐specific CD8^+^ T‐cell‐mediated cancer cell pyroptosis compared with that in scramble‐transfected cells (Figure 5C,D). Importantly, this observation was further confirmed by GZMA ELISA, which revealed that the expression of secreted GZMA was lower in tumor‐specific CD8^+^ T cells after co‐culture with ICAM1‐Huh7^shN1ICD^ cells than in scramble‐transfected Huh7^shN1ICD^ cells (Figure 5E). To further examine this observation, we also stably overexpressed ICAM1 in HepG2^N1ICD^ cells (Figure 5F), which were then used for pyroptotic and cytotoxicity assays with tumor‐specific CD8^+^ T cells. Our data showed that the overexpression of ICAM1 in HepG2^N1ICD^ cells enhanced tumor‐specific CD8^+^ T‐cell‐driven cancer cell pyroptosis, while tumor‐specific CD8^+^ T cells were more effective at killing ICAM1‐overexpressing HepG2^N1ICD^ cells than were empty vector‐transfected HepG2^N1ICD^ cells (Figure 5G,H). Therefore, our data suggest that ICAM1 is the downstream effector of N1ICD‐mediated immunosuppression in HCC cells.

### 2.5 N1ICD Transcriptionally Regulates the Expression of the Transcription Factor YY1 to Modulate ICAM1 Levels in HCC Cells

**Figure 6:**
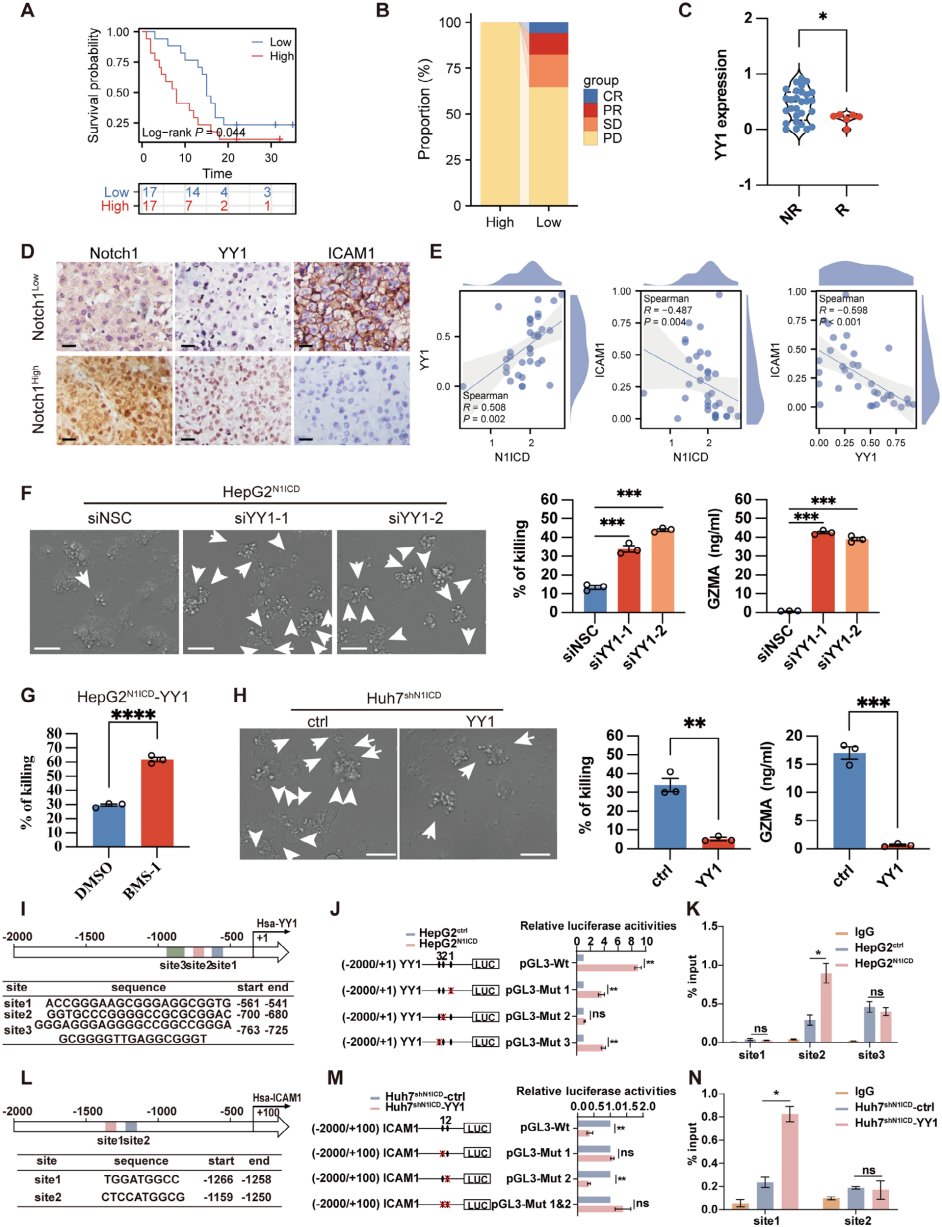
N1ICD transcriptionally upregulates the expression of the transcriptional repressor YY1 to repress ICAM1 levels in HCC cells. A) High expression of YY1 was correlated with poor progression‐free survival, increased recurrence, and a worse immunotherapeutic response in HCC patients after treatment with adjuvant PD‐1/PD‐L1 antibody therapy (*n* = 34 HCC patients, cohort 1). B) Stacked bar showing the therapeutic response to immunotherapy in HCC patients with low or high YY1 expression according to the mRECIST guidelines. C) Violin plot showing the expression level of YY1 in responders and nonresponders. Each sample on the violin plots represents individual patient data (*n* = 34 HCC patients, cohort 1). D) Representative immunohistochemistry images of YY1, ICAM1, and Notch1 staining in tumor sections derived from HCC patients with high or low Notch1 expression are shown. E) In our HCC cohort 1 (*n* = 34), the expression of Notch1 and ICAM1 was negatively correlated (left), the expression of YY1 and Notch1 was positively correlated (middle), and the expression of YY1 and ICAM1 was negatively correlated (right). F) Depletion of YY1 by siRNA in HepG2^N1ICD^ cells enhanced tumor‐specific CD8^+^ T‐cell‐mediated cancer cell pyroptosis and cytotoxicity (*n* = 3 independent experiments). G) LDH release assay of stable N1ICD‐expressing HepG2 cells after transfection with YY1‐targeting siRNA after treatment with tumor‐specific CD8^+^ T cells in the presence of DMSO or BMS‐1 (*n* = 3 independent experiments). H) Overexpression of YY1 in Huh7^shN1ICD^ cells inhibited CD8^+^ T‐cell‐mediated cancer cell pyroptosis (*n* = 3 independent experiments). I) Schematic diagram showing the putative N1ICD binding sites in the human YY1 promoter. J) Luciferase assay of HepG2^N1ICD^/HepG2^ctrl^ cells transfected with a luciferase reporter containing either the full‐length WT YY1 promoter or the mutated YY1 promoter sequence. K) ChIP assays revealed increased binding of N1ICD to its 2^nd^ putative binding site on the YY1 promoter in HepG2^N1ICD^ cells compared with that in HepG2^ctrl^ cells. L) Schematic diagram showing the putative YY1 binding sites in the human ICAM1 promoter. M) Luciferase assay of Huh7^shN1ICD^ cells transfected with a luciferase reporter containing either a full‐length ICAM1 promoter or mutated ICAM1 promoter sequence in the presence of the YY1 overexpression vector or empty control vector (ctrl). N) ChIP assays revealed increased binding of YY1 to its 1^st^ putative binding site on the ICAM1 promoter in Huh7^shN1ICD^ cells transiently transfected with the YY1 overexpression vector compared with empty vector‐transfected cells (*n* = 3 independent experiments). The means ± SEMs are given. ns, nonsignificant difference. *p < 0.05, **p < 0.01, ***p < 0.001, ****p < 0.0001. A) Log‐rank test. E) Spearman correlation study. C,F–H,J,K,M,N) Student's *t*‐test. Scale bars in (F, H) represent 50 µm, D) 100 µm.

Since N1ICD has been shown to regulate target genes by binding to RBPJ/CSL on its promoter,^[^ ^24^ ^]^ we next examined the molecular mechanism whereby N1ICD regulates ICAM1 expression in cancer cells. By performing a transcription factor‐binding site prediction study with the human ICAM1 promoter, vitamin D receptor (VDR) and Yin‐Yang 1 (YY1) were the predicted transcription factors of the human ICAM1 promoter among all three online transcription factor‐binding site prediction tools (i.e., the Cistrome Data Browser,^[^ ^25^ ^]^ PROMO,^[^ ^26^ ^]^ hTFtarget‐liver^[^ ^27^ ^]^; Figure 5L). Further RT‒qPCR and western blot analyses revealed that YY1 was the only transcription factor whose expression was consistently regulated by N1ICD expression in HCC cells (Figure 5M–O). Interestingly, YY1 is known as a transcription factor, the expression of which is strongly correlated with cancer progression, metastasis, and patient prognosis.^[^ ^28^, ^29^ ^]^ However, it remains unclear whether N1ICD regulates ICAM expression via YY1. To examine the role of YY1 in regulating ICAM1 expression, we either stably overexpressed or transiently silenced YY1 in N1ICD‐depleted HCC cells and stable N1ICD‐expressing cells, respectively. Both western blot and RT‒qPCR results indicated that depleting YY1 expression by siRNA upregulated ICAM1 expression in stable N1ICD‐expressing HepG2 cells/Hepa1‐6 cells compared with that in nonsilencing control siRNA‐transfected cells (Figure S5A,B, Supporting Information), whereas aberrant expression of YY1 repressed ICAM1 expression in N1ICD‐stably depleted Huh7/Hepa1‐6 cells compared with that in empty vector‐transfected cells (Figure S5C,D, Supporting Information). To examine the clinical importance of our findings, we performed an immunohistochemistry study of YY1 expression in HCC tumor sections and found that high YY1 expression was correlated with poor PFS and a poor immunotherapeutic response in HCC patients after receiving adjuvant anti‐PD‐1/PD‐L1 antibody therapy (**Figure** **6**A–C). Further clinical studies in both cohort 1 (Figure 6D) and cohort 2 (Figure S5E, Supporting Information) revealed a positive correlation between YY1 and Notch1 expression levels, whereas ICAM1 expression was negatively correlated with both YY1 and Notch1. Additionally, in cohort 2, high expression of YY1 and Notch1 was associated with poor overall survival (OS) (Figure S5F, Supporting Information). Overall, our results indicate that Notch1 might regulate YY1 expression to modulate ICAM1 expression in HCC cells.

We next examined whether YY1 expression confers tumor‐specific CD8^+^ T‐cell cytotoxicity. Our co‐culture experimental results revealed that depletion of YY1 by siRNA in HepG2^N1ICD^ cells had the opposite effect on tumor‐specific T‐cell‐driven cancer cell pyroptosis and pyroptosis‐mediated cytotoxicity as well as GZMA secretion (Figure 6E), whereas overexpression of YY1 in Huh7^shN1ICD^ cells significantly repressed tumor‐specific CD8^+^ T‐cell‐mediated cancer cell pyroptosis and GZMA secretion compared with that in empty vector‐transfected cells (Figure 6F). Importantly, our results showed that inhibition of YY1 by siRNA could enhance the ability of BMS‐1 to promote tumor‐specific CD8^+^ T‐cell cytotoxicity in HepG2^N1ICD^ cells in vitro (Figure 6G). These results suggest that YY1 could be a downstream immunomodulator of the Notch1 signaling pathway.

To explore whether N1ICD regulates YY1 promoter activity directly, we first analyzed the full‐length human YY1 promoter sequence and identified three putative N1ICD binding sites in the human YY1 promoter (Figure 6H). We next transfected HepG2^N1ICD^ cells/HepG2^ctrl^ cells with a luciferase vector containing either a full‐length YY1 promoter sequence or a mutated human YY1 promoter sequence, which indicated that mutating the 2^nd^ putative N1ICD binding site in the human YY1 promoter downregulated its promoter activity in HepG2^N1ICD^ cells (Figure 6I), suggesting that N1ICD regulated the YY1 promoter via this 2^nd^ putative binding site. Further ChIP assays revealed increased N1ICD binding to the 2^nd^ putative binding site of the human YY1 promoter in HepG2^N1ICD^ cells (Figure 6J). To further confirm the role of N1ICD in YY1 promoter activity, we also carried out a promoter study with the mouse YY1 promoter and identified five putative N1ICD binding sites in its promoter. Luciferase assays revealed that mutating the 1^st^/2^nd^/3^rd^ putative N1ICD binding site in the mouse YY1 promoter rescued the decreased promoter activity observed in Hepa‐1‐6^shN1ICD^ cells transfected with a luciferase reporter vector carrying a full‐length YY1 promoter sequence, whereas a ChIP assay revealed decreased binding of N1ICD to the YY1 promoter in Hepa1‐6^shN1ICD^ cells via its 2^nd^ putative binding site only (Figure S6A–C, Supporting Information).

We next examined whether YY1 regulated the expression of ICAM1 by binding to its promoter. Our transcriptional binding site prediction study revealed that the promoter of human ICAM1 contains two YY1 putative binding sites (Figure 6K). We therefore generated a series of luciferase reporter vectors carrying either a full‐length ICAM1 promoter sequence or a site‐mutated promoter sequence, which were then used to perform luciferase assays with Huh7^shN1ICD^ cells. Our results indicated that mutation of the 1^st^ putative YY1 binding site prohibited YY1‐mediated repression of ICAM1 promoter activity in Huh7^shN1ICD^ cells transfected with the YY1 overexpression vector compared with that in cells transfected with a luciferase reporter containing a full‐length WT ICAM1 promoter sequence (Figure 6L). This observation was further confirmed by a ChIP experiment using a YY1 antibody, which suggested that YY1 bound to its 1^st^ putative site on the ICAM1 promoter in HCC cells (Figure 6M). Overall, our results demonstrated that N1ICD transcriptionally regulated ICAM1 expression through YY1 in HCC cells.

### 2.6 Depletion of YY1 by siRNA Enhances PD‐L1 Antibody Efficacy Against Orthotopic Liver Tumor Growth Without Causing Severe Side Effects

**Figure 7:**
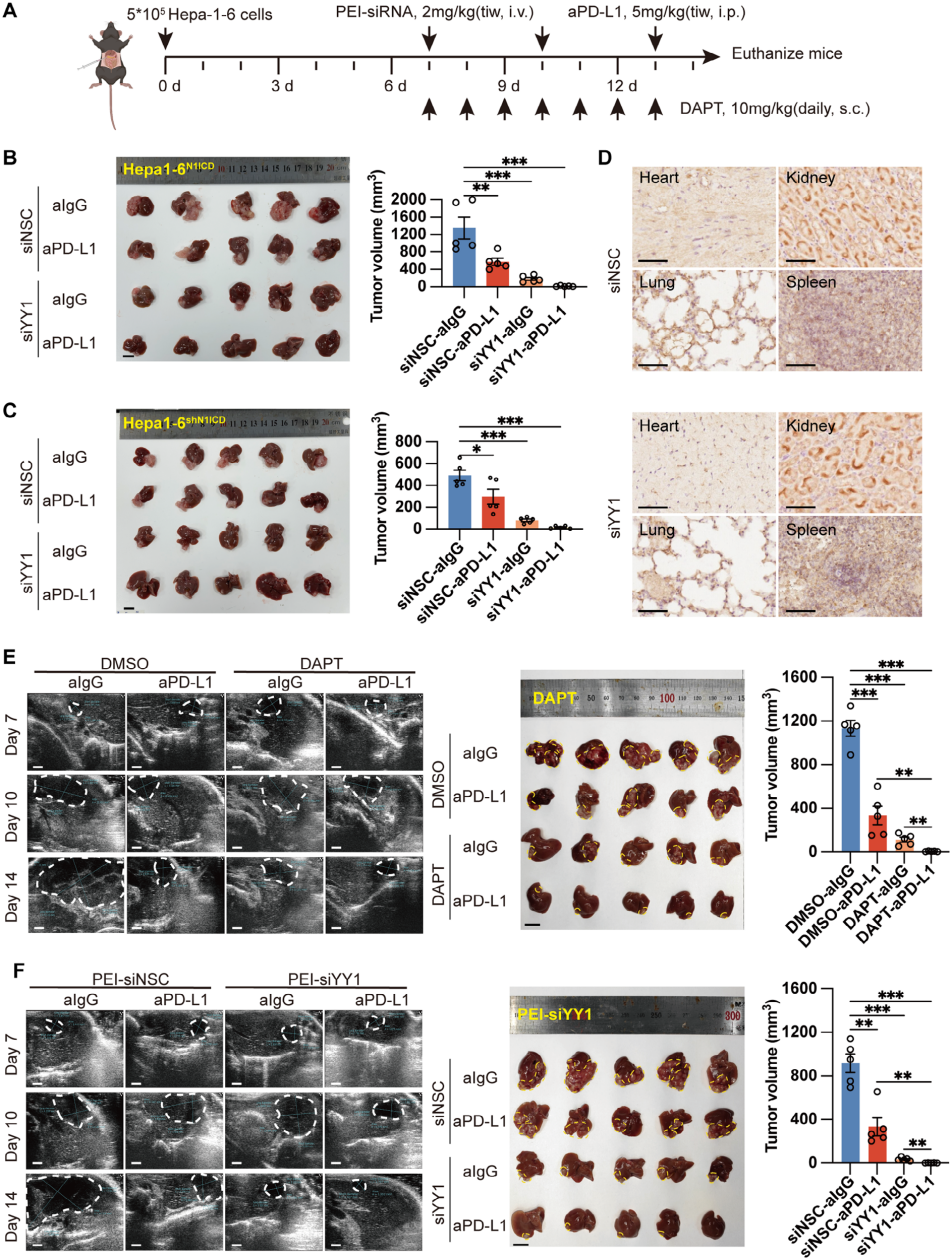
Combined treatment with the PEI‐siYY1 complex and PD‐L1 antibody significantly repressed orthotopic HCC tumor growth without causing any adverse side effects. A) Schematic diagram of the combined treatment of PD‐L1 monoclonal antibody and DAPT or PEI‐siYY1 complex in the HCC mouse orthotopic model (created with BioRender.com). Seven days after orthotopic Hepa‐1‐6 cell injection, the tumor‐bearing mice were treated with either DAPT (10 mg kg^−1^, s.c.) for 7 consecutive days or the PEI‐siYY1 complex (2 mg kg^−1^, i.v.) every 3 days for up to 7 days together with the IgG control or PD‐L1 antibody (aPD‐L1) (5 mg kg^−1^, i.p.) every 3 days for up to 3 times. Tumor growth was monitored via in vivo ultrasound imaging every 3 days for a total of 3 days (*n* = 5 mice per group). B,C) Representative gross Hepa1‐6^N1ICD^ (B)‐ or Hepa1‐6^shN1ICD^ (C)‐derived tumor images from each treatment group are shown (left), and the final tumor volumes are shown in a bar chart (right). D) Representative images of immunohistochemical staining for YY1 in the heart, lung, spleen, and kidney. E) Representative ultrasound scanning images (left), gross tumor images (middle), and final tumor volume quantification (right) of Hepa1‐6 orthotopic tumor models treated with DAPT combined with an anti‐PD‐L1 antibody (or solvent control) are shown (*n* = 5 mice per group). F) Representative ultrasound scanning images (left), gross tumor images (middle), and final tumor volume quantification (right) of Hepa1‐6 orthotopic tumor models treated with PEI‐siYY1 combined with an anti‐PD‐L1 antibody (or solvent control) are shown (*n* = 5 mice per group). The means ± SEMs are given. **p < 0.01, ***p < 0.001, ****p < 0.0001. B,C,E,F) Student's *t*‐test. The scale bars in (E) represent 1 mm, (B,C,E,F) 1 cm, and (D) 50 µm.

Although a Notch1 inhibitor can enhance immune checkpoint inhibitor efficacy in vivo, its treatment alone has been shown to cause adverse side effects in some clinical trials. Since we have shown that N1ICD reduces ICAM1 expression to block immune checkpoint inhibitor efficacy by increasing YY1 expression in vitro, we next investigated whether inhibition of YY1 could enhance anti‐PD‐L1 efficacy without causing severe toxic side effects in vivo. We first designed and labeled YY1‐targeting siRNA (siYY1)/nonsilencing control siRNA (siNSC) with 2′OMe modification, which was then mixed with an in vivo/jet polyethyleneimine (PEI) regent (i.e., the PEI‐siYY1 or PEI‐siNSC complex) and used for animal experiments (**Figure** **7**A). Next, C57BL/6 mice were orthotopically implanted with N1ICD‐knockdown or N1ICD‐overexpressing Hepa1‐6 cells. The mice bearing orthotopic tumors were then treated with 2 mg kg^−1^ PEI‐siYY1/‐siNSC complex every three days for up to 3 times, together with 5 mg kg^−1^ anti‐PD‐L1 or IgG control antibody every three days for up to 3 times. Critically, PEI‐siYY1 significantly enhanced the therapeutic efficacy of anti‐PD‐L1 therapy in both N1ICD‐knockdown and N1ICD‐overexpressing orthotopic models (Figure 7B,C). Safety assessments of major organs (heart, lung, kidney, and spleen) revealed no off‐target effects of PEI‐siYY1, supporting its therapeutic specificity (Figure 7D). In a parallel therapeutic approach, tumor growth was monitored via an in vivo ultrasound imaging system. Mice bearing tumors were treated with either 10 mg kg^−1^ d^−1^ of the Notch1 inhibitor DAPT/DMSO solvent (Figure 7E) or 2 mg kg^−1^ PEI‐siYY1/‐siNSC complex every 3 days for up to three times (Figure 7F) together with 5 mg kg^−1^ anti‐PD‐L1 or IgG control antibody every three days for up to three times. Strikingly, treatment with either PEI‐siYY1 or DAPT significantly enhanced the efficacy of the PD‐L1 antibody against HCC orthotopic tumor growth compared with that of the groups treated with the anti‐PD‐L1 antibody alone (Figure 7E,F). Further immunohistochemistry revealed that treatment with either PEI‐siYY1 or DAPT reduced YY1 expression and increased ICAM1 expression and CD8^+^ T‐cell numbers in orthotopic Hepa‐1‐6 liver tumors compared with those in the groups treated with either the DMSO solvent, the PEI‐siNSC complex, or the anti‐PD‐L1 antibody alone (Figure S7A, Supporting Information). Periodic acid‐Schiff (PAS) staining of the small intestine revealed that in mice treated with DAPT and the anti‐PD‐L1 antibody, the intestinal mucosa developed secretory metaplasia, as indicated by a significant increase in the number of goblet cells (Figure S7A, Supporting Information). Nevertheless, combined treatment with DAPT and an anti‐PD‐L1 antibody had strong adverse effects, including diarrhea and significant weight loss, in HCC orthotopic‐bearing mice, whereas these severe toxicity effects were not observed in the mice treated with the PEI‐siYY1 complex and PD‐L1 antibody combination therapy (Figure S7B,C, Supporting Information).

**Figure 8:**
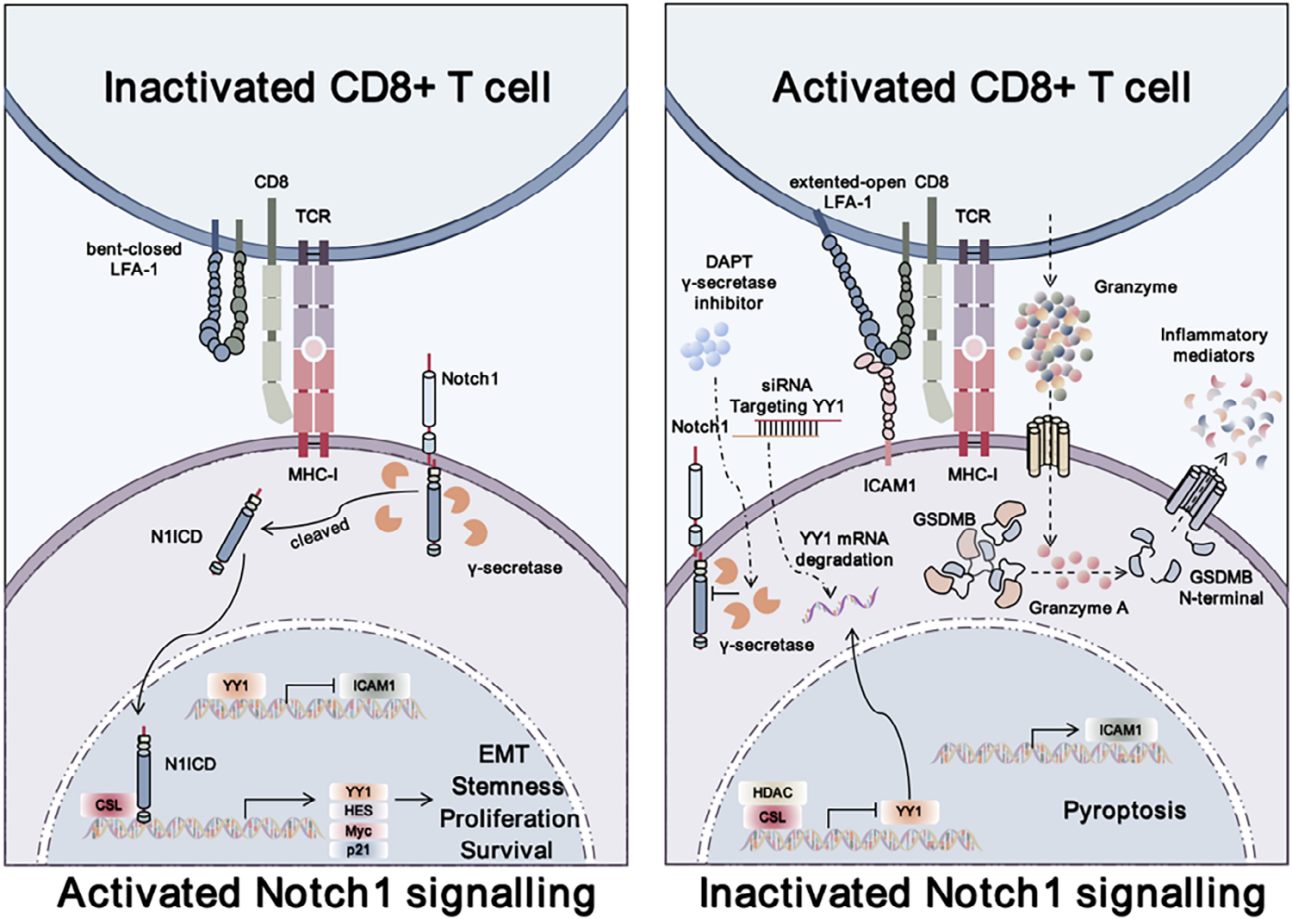
Schematic diagram showing the role of the Notch1‒YY1‒ICAM1 signaling axis in modulating tumor immune escape. Notch1 overexpression transcriptionally upregulated the expression of YY1, which in turn repressed ICAM1 expression to prohibit CD8^+^ T‐cell‐derived granzyme A‐driven cancer cell pyroptosis and its mediated cytotoxicity.

Collectively, our data demonstrated that high Notch1 expression was correlated with poor immunotherapeutic response and progression‐free survival in HCC patients. Mechanistically, increased N1ICD expression upregulated the expression of the transcriptional repressor YY1 to repress ICAM1 expression, which mediated CD8^+^ T‐cell‐derived granzyme‐driven cancer cell pyroptosis, leading to tumor immune escape (**Figure** **8**A). Importantly, we showed that depletion of YY1 by siRNA, rather than depletion of Notch1, enhanced immunotherapy efficacy in HCC without causing significant adverse side effects in vivo, providing an improved cancer treatment strategy for advanced HCC patients.

## 3 Discussion

Our results revealed that increased N1ICD expression upregulates the expression of the transcriptional repressor YY1 to repress ICAM1 expression at the transcriptional level, which in turn prohibits tumor‐specific CD8^+^ T‐cell toxicity and granzyme‐mediated GSDMB‐driven cancer cell pyroptosis. Importantly, targeting YY1, rather than Notch1, increases PD‐L1 antibody treatment efficacy in an orthotopic HCC tumor model without causing adverse side effects. These results call for us to reconsider our strategy for targeting the Notch1 signaling pathway in HCC treatment.

Immunotherapy has provided limited clinical benefits in advanced HCC patients, probably because of its complex immunosuppressive tumor microenvironment; however, the underlying molecular mechanism behind this phenomenon remains unclear.^[^ ^30^, ^31^ ^]^ We and others have shown that the Notch signaling pathway plays an important role in modulating HCC development and progression, while increased Notch1 signaling is known to correlate with increased immunosuppressive cell infiltration in the tumor microenvironment.^[^ ^8^, ^32^ ^]^ In animal models, depletion of cancer cell‐Notch1 improved anti‐PD‐L1 efficacy in melanoma and head and neck cancer through a poorly studied mechanism.^[^ ^11^, ^33^ ^]^ In the clinic, Notch signaling has been shown to predict the immunotherapeutic response in patients with small cell lung cancer,^[^ ^34^ ^]^ but its role in HCC has not been explored. In this study, we first showed that high Notch1 expression is associated with poor progression‐free survival, increased tumor recurrence and a worse immunotherapeutic response in HCC patients after receiving adjuvant anti‐PD1/PD‐L1 therapy. In addition, our data show that depletion of N1ICD enhances PD‐L1 antibody efficacy and CD8^+^ T‐cell infiltration in a mouse orthotopic liver tumor model. Consistently, inhibition of Notch1 has been linked with CTL infiltration in a C57BL/6 melanoma model.^[^ ^20^, ^33^ ^]^ We next showed that cancer cell‐N1ICD prohibits tumor‐specific T‐cell cytotoxicity in vitro and in vivo, while depletion of N1ICD also enhances the effectiveness of combined adoptive cell transfer therapy and BMS‐1 against human HCC subcutaneous tumor growth. These results suggest that targeting cancer cell‐Notch1 signaling can be an effective method to increase immunotherapy efficacy. However, several clinical studies have shown that Notch1 inhibitors (GSIs: crenigacestat^[^ ^35^, ^36^ ^]^ and RO4929097^[^ ^37^ ^]^), even at their maximum tolerated doses, cannot exert sufficient antitumor effects, whereas the off‐target side effects and toxicity of Notch1 inhibitors have raised concerns about their clinical application. Indeed, our animal model also revealed that the administration of the Notch1 inhibitor DAPT can enhance PD‐L1 antibody efficacy against HCC orthotopic tumor growth but can cause severe adverse side effects. Taken together, these findings suggest that targeting the downstream immunomodulator of Notch1 signaling, rather than Notch1 itself, could be a better strategy for enhancing cancer immunotherapy efficacy.

By performing RNA‐sequencing analysis of N1ICD‐depleted Huh7 cells and scramble‐transfected cells, together with the results of clinical studies, we showed that ICAM1 expression is most significantly correlated with Notch1 expression in HCC. Despite evidence indicating that intercellular adhesion molecule‐1 (ICAM1) serves as a neoplastic stem cell marker associated with progression and metastasis in melanoma, breast cancer, thyroid cancer, and nasopharyngeal carcinoma, accumulating evidence underscores its critical role in CD8+ T‐cell activation. As a costimulatory molecule, ICAM1 binds to lymphocyte function‐associated antigen‐1 (LFA‐1) on lymphocyte surfaces, mediating immune cell migration, infiltration, and immunological synapse formation, thereby potentiating antitumor immunity. Recent research has demonstrated that cancer cells inhibit the binding of ICAM1 to LFA‐1 via PSGL‐1, consequently suppressing macrophage phagocytosis, which further substantiates the importance of ICAM1 as a pivotal effector molecule in antitumor immune responses.^[^ ^38^, ^39^, ^40^ ^]^ Additionally, its expression is required for the surveillance of cytotoxic T lymphocytes. Indeed, we showed that depleting ICAM1 with a short hairpin RNA (shRNA) or neutralizing antibody prohibits tumor‐specific CD8^+^ T cells from inducing pyroptosis and cytotoxicity in N1ICD‐depleted HCC cells, while both luciferase and chromatin immunoprecipitation (ChIP) assays revealed that N1ICD transcriptionally upregulates the expression of the transcriptional repressor YY1, which in turn represses ICAM1 promoter activity and transcription. Clinically, we showed that the expression of YY1 and Notch1 was positively correlated in our HCC patient cohort, whereas the expression of YY1 was negatively correlated with ICAM1 expression in the same HCC cohort. Importantly, YY1 expression also affects the immunotherapeutic response and prognosis of HCC patients. Consistently, the expression of YY1 has been linked with cancer growth and progression in other cancer types via a poorly studied mechanism. Thus, our results demonstrated for the first time that cancer cell‐specific Notch1 expression modulates cancer immunotherapy efficacy by increasing YY1‐mediated suppression of ICAM expression.

Recent studies have indicated that cytotoxic T cells can secrete granzymes to stimulate GSDMB‐positive cancer cells to undergo pyroptosis,^[^ ^18^ ^]^ whereas the activation of pyroptosis has been shown to promote an antitumor immune response and PD‐L1 treatment efficacy, likely through the release of inflammatory factors (i.e., IL‐18 and IL‐1β).^[^ ^23^, ^41^ ^]^ Indeed, we showed that, compared with scramble‐transfected cells, tumor‐specific CD8^+^ T cells are more effective at inducing N1ICD‐depleted HCC cells to undergo GSDMB‐driven pyroptosis, whereas stable N1ICD‐expressing HCC cells are resistant to tumor‐specific CD8^+^ T‐cell‐driven GSDMB‐dependent pyroptosis and its mediated cytotoxicity. These results suggest that N1ICD expression may suppress tumor‐specific CD8^+^ T‐cell cytotoxicity against cancer cells, likely by inhibiting granzyme‐mediated pyroptosis. Importantly, we show that the inhibition of granzymes by inhibitors prohibits tumor‐specific CD8^+^ T‐cell‐driven cancer cell pyroptosis and pyroptosis‐mediated cytotoxicity, whereas the silencing of GSDMB in N1ICD‐depleted HCC cells also suppresses cancer cell pyroptosis and cytotoxicity, suggesting that tumor‐specific CD8^+^ T‐cell‐derived granzyme‐driven cancer cell pyroptosis may contribute to the cytotoxicity of GSDMB in cancer cells. We next examined the role of ICAM1 in tumor‐specific CD8^+^ T‐cell‐mediated pyroptosis and found that the overexpression of ICAM1 in N1ICD‐expressing HCC cells enhances tumor‐specific CD8^+^ T‐cell‐driven cancer cell pyroptosis and cytotoxicity, whereas the silencing of ICAM1 in N1ICD‐depleted HCC cells has the opposite effect. Overall, we show that ICAM1 is a downstream immunomodulator of Notch1 signaling, which can affect tumor‐specific CD8^+^ T‐cell cytotoxicity and drive cancer cell pyroptosis.

Since we have shown that N1ICD regulates YY1 expression to suppress ICAM1 expression in HCC cells, we next examined whether targeting YY1 expression can be a safe and effective method to increase PD‐L1 antibody efficacy in vivo. By using an in vivo siRNA delivery system, we showed that combined treatment with PEI‐siYY1 and an anti‐PD‐L1 antibody strongly repressed the growth of orthotopic liver tumors without causing severe adverse side effects, which were observed in the group treated with the Notch1 inhibitor DAPT and an anti‐PD‐L1 antibody. Consistently, YY1 has been shown to determine cancer growth and progression in different cancer types. Therefore, genetic modulation of YY1 may be a safe and effective way to promote PD‐L1 antibody efficacy in vivo, but further investigations should be carried out to examine whether the synergistic suppressive effect of YY1 depletion and PD‐L1 antibodies on tumor growth may be due to the activation of tumor‐specific CD8^+^ T‐cell‐secreted granzyme‐driven cancer cell pyroptosis.

In conclusion, we discovered a novel mechanism of tumor immune escape involving the activation of Notch1 signaling and its target gene YY1, which reduces immunotherapy efficacy by suppressing cancer cell‐ICAM1‐driven T‐cell activation and its mediated cytotoxicity against cancer cells, providing a safe and effective therapeutic target for combination immunotherapy strategies.

## 4 Experimental Section

### Human Clinical Specimens

Two patient cohorts with HCC were included in our study. Cohort 1 included 34 paired paraffin‐embedded normal adjacent tissue and tumor samples derived from postoperative recurrent HCC patients who received at least four cyclical adjuvant anti‐PD1/PD‐L1 monoclonal antibody treatments (i.e., once every 3 weeks via intravenous injection) at Sun Yat‐Sen Memorial Hospital, Sun Yat‐sen University, between 2017 and 2020. The immunotherapeutic response and recurrence of patients were monitored via computed tomography (CT) imaging or by measuring the serum AFP level according to the mRECIST guidelines.^[^ ^42^ ^]^ Cohort 2 included 67 paraffin‐embedded tumor samples derived from HCC patients who underwent surgery without receiving any immunotherapy at Sun Yat‐Sen Memorial Hospital. All the samples included in this study were anonymously coded in accordance with local ethical guidelines with written informed consent and a protocol approved by the Ethical Review Committee of Sun Yat‐sen Memorial Hospital. Human hepatocellular carcinoma (HCC) and clinical data were obtained from the Ethical Review Committee of Sun Yat‐sen Memorial Hospital (SYSEC‐KY‐KS‐2021‐306), with signed informed consent from patients and ethical committee approval. All animal procedures were approved by the Institutional Animal Care and Use Committee of South China University of Technology (2022004).

### Small Interfering RNA, Plasmids, and Transfection

To generate stable N1ICD‐expressing cells, HepG2 cells were stably transfected with pCDH‐CMV‐GFP‐puro‐N1ICD (GenePharma, Shanghai) or a control empty plasmid (GenePharma, Shanghai) and subjected to selection with 2 µg mL^−1^ puromycin (Beyotime, Cat. no. ST551‐10 mg) as previously described. To knock down N1ICD expression, Huh7 or MHCC‐97H cells were stably transfected with pCDH‐CMV‐GFP‐puro‐shN1ICD (GenePharma, Shanghai) or scramble control plasmid (GenePharma, Shanghai). To construct stable ICAM1 knockdown cells, Huh7‐shN1ICD cells were stably transfected with pLKO.1‐Neo‐shICAM1 (IGE BIOTECHNOLOGY LTD, Guangzhou) or scramble control plasmid (IGE BIOTECHNOLOGY LTD, Guangzhou) and selected with 800 µg mL^−1^ neomycin (Cytiva, Cat. no. 0215878291). To construct stable ICAM1‐expressing cells, HepG2‐N1ICD cells were stably transfected with pLKO.1‐NEO‐ICAM1 (IGE Biotechnology Ltd., Guangzhou) and selected with 800 µg mL^−1^ neomycin (Cytiva, Cat. no. 021 587 8291). For the generation of GSDMB stable knockdown cells, HepG2 and Huh7‐shN1ICD cells were first stably transfected with pLKO.1‐shGSDMB‐Neo (IGE BIOTECHNOLOGY LTD, Guangzhou) or scramble control plasmid (IGE BIOTECHNOLOGY LTD, Guangzhou), which were then subjected to 1000 µg mL^−1^ neomycin (Cytiva, Cat. no. 021 587 8291) selection. For small interfering RNA transfection, HepG2‐N1ICD and Hepa‐1‐6 cells were transfected with YY1‐targeting siRNA (IGE BIOTECHNOLOGY LTD, Guangzhou) or nonsilencing siRNA control (IGE BIOTECHNOLOGY LTD, Guangzhou) via Lipofectamine 3000 (Invitrogen, Cat. no. L3000015). For YY1 plasmid transfection, Huh7^shN1ICD^ or Hepa1‐6^shN1ICD^ cells were transiently transfected with a YY1 overexpression plasmid (IGE Biotechnology Ltd., Guangzhou) or an empty control plasmid (IGE Biotechnology Ltd., Guangzhou) via Lipofectamine 3000 (Invitrogen, Cat. no. L3000015). The sequences of the siRNAs are listed in Table S2 (Supporting Information).

### Real‐Time Quantitative PCR

Total RNA was extracted via an RNA extraction kit (ESscience, Cat. no. PC001), while the reverse transcription process was completed via a PrimeScript RT Reagent Kit (TAKARA, Cat. no. RR036A), in accordance with the manufacturer's protocols. The obtained cDNA was subjected to real‐time quantitative PCR with a Green Premix Ex Taq II kit (TAKARA, Cat. no. RR820A) via a Roche LightCycler 480II machine. The final cycle time value (Ct) of a target gene was normalized to that of GAPDH as an internal reference. The relative expression of the genes was represented by the 2^‐ΔΔCt method^, and the control group was defined as 1. The sequences of the primers used for quantitative real‐time PCR are listed in Table S3 (Supporting Information).

### Western Blotting

The total protein of the cells was extracted with RIPA lysis buffer (CoWin Biosciences, Cat. no. CW2333S) containing 1× protease inhibitor (CoWin Biosciences, Cat. no. CW2200S) and 1× phosphatase inhibitor (CoWin Biosciences, Cat. no. CW2383S) on ice. The supernatant was aspirated after centrifugation and quantified via the PIERCE BCA PROTEIN ASSAY kit (Invitrogen, Cat. no. 23 227). Before the protein sample was loaded, 5× sodium dodecyl sulfate‐sample buffer was added to the supernatant, which was then transferred to a thermostat water bath at 95 °C for 10 min. Equal amounts of protein samples were loaded in each lane and separated by sodium dodecyl sulfate‒polyacrylamide gel electrophoresis (SDS‒PAGE). The concentration of the SDS‒PAGE gel used was dependent on the molecular weight of the target protein. The samples were transferred to polyvinylidene difluoride (PVDF) membranes and blocked with 5% BSA in Tris‐buffered saline with Tween (TBST) at room temperature for 1 hour. After blocking, the membrane was incubated with a primary antibody overnight at 4 °C. After being washed 3 times with TBST, the membrane was incubated with an HRP‐conjugated secondary antibody (Cell Signaling Technology, Cat. no. 7074S) at RT for 1 h, and protein expression was detected via enhanced chemiluminescence (ECL) (Fudebio, Cat. no. FD8020) and MiniChemi (SAGECREATION, Beijing, China). The primary antibody details are given in Table S4 (Supporting Information).

### Immunohistochemical (IHC) Staining

The IHC staining and analysis methods were performed as previously described.^[^ ^8^, ^43^ ^]^ Briefly, the paraffin‐embedded tissue sections were first dewaxed with xylene, rehydrated with a concentration gradient of ethanol, and subsequently subjected to antigen retrieval buffer in boiling conditions for 10 min. The slides were first blocked with 1% normal goat serum (NGS) for 30 min at room temperature and then incubated with primary antibody in a humidified chamber overnight at 4 °C. After incubation, the slides were first washed in PBS three times and then incubated with an HRP‐conjugated secondary antibody (Dako, Cat. no. K5007) at 37 °C for 30 min. Finally, the slides were washed with PBS 3 times, incubated with DAB (Dako, Cat no. K5007), and counterstained with hematoxylin. The details of the primary antibodies used in this study are listed in Table S4 (Supporting Information).

The IHC images were captured via a phase contrast microscope (Nikon, Japan) and were assessed in a double‐blinded manner by two independent pathologists.^[^ ^8^ ^]^ The expression level of tumor‐N1ICD was scored by measuring its nuclear and cytoplasmic expression in cancer cells. The intensity of staining was defined as 0 (negative), 1 (weak), 2 (medium), or 3 (strong), and the percentage of positive cancer cells was determined in the whole tissue section. The N1ICD score was calculated by multiplying the staining intensity of N1ICD by the percentage of NI1CD‐positive cancer cells within the whole tissue section. The YY1 and ICAM1 expression score was defined as the ratio of positively immunostained cancer cells to hematoxylin‐positive cancer cells. The score of CD8 intensity was defined as the proportion of CD8‐positive cells in the total number of hematoxylin‐positive cells.

### Cell Culture

Human HCC cell lines, including Huh7 (RRID: CVCL_0336), MHCC‐97H (RRID: CVCL_4972), and HepG2 (RRID: CVCL_0027), were purchased from the Cell Bank of the Chinese Academy of Sciences (Shanghai, China). The mouse HCC cell line Hepa‐1‐6 (RRID: CVCL_0327) was also purchased from the Cell Bank of the Chinese Academy of Sciences. All the cell lines were cultured in high‐glucose DMEM supplemented with 10% fetal bovine serum (FBS) and 1% penicillin/streptomycin at 37 °C in a 5% CO_2_ incubator.

### RNA‐Sequencing Analysis

Total RNA was extracted from the cell samples by using TRIzol reagent (TAKARA, Cat. no. 9109) according to the manufacturers’ instructions. In brief, the samples were first digested with DNase to remove DNA and then subjected to magnetic beads with oligo (dT) incubation to enrich the mRNA content, after which they were used to synthesize cDNAs. After the constructed cDNA library was analyzed via an Agilent 2100 Bioanalyzer, the Illumina HiSeqTM 2500 sequencer was subsequently used to sequence and generate 125‐bp or 150‐bp paired‐end data. After the quality inspection, the Illumina sequencer was used for sequencing. Transcriptome sequencing and analysis were conducted by OE Biotech Co., Ltd. (Shanghai, China). The known reference gene sequences and annotation files were used as a database, and a sequence similarity comparison method was adopted to identify the expression abundance of each protein‐coding gene in each sample. Htseq‐count software was used to obtain the number of reads compared with the number of protein‐coding genes in each sample as previously described, while Cufflinks software was used to calculate the FPKM value of protein‐coding gene expression. A two‐class comparison between groups was performed to define differentially expressed genes (DEGs). The threshold at a fold change of 1.5 was used to define the upregulated or downregulated genes, with a *p*‐value < 0.05 indicating a statistically significant fold change. DEGs were subjected to GO enrichment analysis and pathway analysis via the Kyoto Encyclopedia of Genes and Genomes (KEGG) database to determine their functions.

### Generation of Cancer Cell Lysate Pulsed with Dendritic Cells

Peripheral blood mononuclear cells (PBMCs) derived from healthy donors were isolated with Ficoll (Stemcell, Cat. no. 0 7861) by performing density gradient centrifugation at room temperature. Monocytes were isolated from PBMCs by using anti‐CD14 magnetic beads according to the manufacturer's protocol (Miltenyi Biotec, Cat. No. 130‐050‐201). For the generation of mature dendritic cells (DCs), monocytes were cultured and induced in conditioned medium (RPMI‐1640 medium containing 10% FBS, 1% penicillin/streptomycin, 55 µm β‐mercaptoethanol, 500 U mL^−1^ IL‐4 (PeproTech, Cat. no. 200‐04‐20), 800 U mL^−1^ GM‐CSF (PeproTech, Cat. no. 300‐03‐20)) for 5 days, and 20 ng mL^−1^ TNF‐α (Sino Biological, Cat. no. 10602‐R10N1‐200) was then added to the culture for 2 days to induce DC maturation. The mature DCs were finally incubated with 50 µg mL^−1^ cancer cell lysate (i.e., total antigens) for 24 h to generate cancer cell‐specific DCs.^[^ ^44^, ^45^ ^]^

### Generation of DC‐Induced Cancer Cell‐Specific CD8^+^ T Cells

After monocytes were obtained from the PBMCs, the CD14‐negative cells were incubated with anti‐CD8 magnetic beads (Miltenyi, Cat. no. 130–045–201) according to the manufacturer's protocol to obtain CD8^+^ T cells. The cancer cell‐specific DCs were used as stimulators and were co‐cultured with CD8^+^ T cells in conditioned medium (RPMI‐1640 medium supplemented with 10% FBS, 1% penicillin/streptomycin, 55 µm β‐mercaptoethanol, and 10 ng mL^−1^ IL‐2) at a ratio of 10:1 for 10 days to obtain DC‐primed tumor‐specific CD8^+^ T cells.^[^ ^45^ ^]^

### Cancer Cell Antigen Preparation

A total of 1 × 10^8^ HCC cells were first trypsinized from the culture plates, which were then centrifuged, resuspended in PBS to 10^7^ mL^−1^, and finally transferred to a cryopreservation tube. The tube was quickly frozen for 10 min in liquid nitrogen and then transferred to a thermostat water bath at 37 °C for 10 min. These procedures were repeated for more than five cycles. The tube was finally centrifuged at 3000 rpm at 4 °C for 5 min, and the supernatant was the cancer cell antigen that could be used to pulse the dendritic cells.^[^ ^46^ ^]^

### Cytotoxic T‐Cell‐Mediated Killing Assays

To assay killing mediated by T cells, 5000 cancer cells (target cells) were seeded onto a 96‐well cell culture plate. After 8 h, 10 µg mL^−1^ mitomycin C (APExBIO, Cat. no. A4452‐5 mg) was added to each well, and the mixture was incubated for 30 min at 37 °C and then washed twice with PBS. Non‐DC‐primed/tumor‐specific CD8^+^ T cells (effector cells) were added to target cells at various effector/target cell ratios (E/T = 1:1, 10:1, 20:1, 40:1) and co‐cultured for 12 h. Target cells not co‐cultured with CD8^+^ T cells were used as the control group to calculate the spontaneous release and maximum release of lactate dehydrogenase (LDH). After 12 h of co‐culture, the supernatant was aspirated, and the release of LDH was detected via an LDH Cytotoxicity Assay Kit (Yeasen, Cat. no. 40209ES76) according to the manufacturer's protocol. The percentage of killing by T cells was expressed as the relative amount of LDH, which is indicative of target cell death, and was calculated as follows: (LDH release from the co‐culture supernatant − target cell spontaneous release − effector cell spontaneous release)/(target cell maximum release − target cell spontaneous release) × 100%. A blank control (only culture medium) was subtracted from the above LDH value. To inhibit the release of granzymes, effector/target co‐culture was performed in the presence of 5 mM EGTA (ethylene glycol‐bis(β‐aminoethyl ether)‐N,N,N′,N′‐tetraacetic acid) (Macklin, Cat. no. E885919‐100 ml)[21]. To inhibit the activity of granzymes, lymphocytes were pretreated with 100 µM DCI (3,4‐dichloroisocoumarin) (Macklin, Cat. no. D909910‐5 mg) at 37 °C for 1 h, washed twice with PBS and then added to the target cells[51]. The final concentration of DMSO (dimethyl sulfoxide) (Cytiva, Cat. no. 021 960 5580) used during DCI treatment was ≈1%.

### Neutralization Assay

Cancer cells were seeded onto a culture plate for 8 h, which was then incubated with 20 µg mL^−1^ ICAM1‐blocking monoclonal antibody (eBioscience, Cat. no. 14‐0549‐82) at 37 °C for 30 min to neutralize ICAM1 on the cell surface and then washed twice with PBS.

### Flow Cytometry Analysis

To determine the activation of T cells after co‐culture, 2 × 10^4^ cancer cells were seeded onto a 24‐well cell culture plate. Eight hours later, 10 µg mL^−1^ mitomycin C (APExBIO, Cat. no. A4452‐5 mg) was added to each well, and the samples were incubated at 37 °C for 30 min and then washed twice with PBS. CD8^+^ T cells were added to the target cells at an E/T ratio of 10:1 and co‐cultured for 12 h. Brefeldin A (BFA) (1 µL; Biolegend, Cat. no. 420 601) was added to the co‐culture for 4 h. Finally, the suspended cells were collected and washed twice with FACS buffer (1% BSA in PBS). Afterward, the cells were incubated with a fluorescently labeled primary antibody (CD8, CD107a) at 4 °C in the dark for 30 min. After incubation, the cells were washed twice and resuspended in a final volume of 200 µL of FACS buffer. The primary antibody information is listed in Table S4 (Supporting Information).

### Predicting the Transcription Factor‐Binding Sites on Target Gene Promoters

The promoter sequences of human/murine YY1 and ICAM1 were obtained from the EPD website (https://epd.epfl.ch/EPDnew_database.php). The binding sites of transcription factors and promoters were predicted via the following web tools according to the developer's instructions: CistromeDB database (http://dbtoolkit.cistrome.org/), PROMO database (http://alggen.lsi.upc.es/cgi‐bin/promo_v3/promo/promoinit.cgi?dirDB=TF_8.3), JASPAR (https://jaspar.genereg.net/), and hTFtarget‐liver database (http://bioinfo.life.hust.edu.cn/hTFtarget#!/). The predicted binding sites are shown in the supplementary materials. The genes predicted to bind to the ICAM1 promotor through databases are listed in Table S5 (Supporting Information).

### Dual‐Luciferase Reporter Assay

The cells were seeded onto 24‐well cell culture plates, which were first transfected with siRNA targeting our gene of interest for 24 hours and then cotransfected with 500 ng pRL‐TK Renilla, pGL3‐promoter‐luci reporter vectors, or basic vector plasmids (pGL3/pRL‐TK = 10:1). pRL‐TK was used as an internal control. After 48 h, the cells were lysed for the dual luciferase reporter experiment, which was performed in accordance with the manufacturer's protocol (Promega, Cat. no. E1960). The promoter activity was defined as the ratio of firefly/Renilla luciferase intensity, where the firefly luciferase activity was normalized to the Renilla luciferase activity. The predicted sites are listed in Tables S6 and S7 (Supporting Information).

### Chromatin Immunoprecipitation (ChIP)

ChIP experiments were performed by using a ChIP assay kit (Beyotime, Cat. no. P2078) according to the manufacturer's instructions. In brief, 3 × 10^6^ cells were fixed with 1% paraformaldehyde at 37 °C for 15 min for cross‐linking. After lysis, an ultrasonic disruptor (Bioruptor PLUS, Canada) was used to break the DNA genome into approximately 400–800‐bp protein‒DNA complexes, which were then incubated with N1ICD antibody (Cell Signaling Technology, Cat. no. 3608s), YY1 antibody (Cell Signaling Technology, Cat. no. 46395S) or IgG control antibody (Cell Signaling Technology, Cat. no. 3900S) as a control. The enriched chromatin was purified to obtain DNA, which was used for RT‒PCR analysis, and the Ct values were normalized to the DNA input. The sequences of primers used for the ChIP analysis are listed in Tables S8 and S9 (Supporting Information).

### Enzyme‐Linked Immunosorbent Assay (ELISA)

ELISA kits were used to detect the expression level of granzyme A (GZMA) (Signalway Antibody, Cat. no. EK2015) in the culture medium of target/effector co‐cultures according to the manufacturer's instructions.

### Subcutaneous HCC Tumor Model

N1ICD‐depleted Huh7 (Huh7^shN1ICD^) cells (3 × 106) were suspended in 100 µL of Matrigel (Corning, Cat. no. 354 234) in PBS (the ratio of Matrigel/PBS = 1:1) and injected into the right flanks of 4‐week‐old female NOD/SCID mice. The tumors were measured by using a caliper, and the tumor volume was calculated as follows: (Volume = a×b×b/2), where “a” and “b” represent the perpendicular short and long diameters, respectively. The mouse weights and tumor sizes were monitored every 3 days for 22 days. Once the tumors reached 50 mm^3^, the mice were randomly divided into two groups, which were given DC‐pulsed CD8^+^ T cells (i.e., adoptive cell transfer, ACT) in the presence of an anti‐PD‐L1 monoclonal antibody (aPD‐L1) (BioXCell, Cat. no. BP0101‐25MG) or an anti‐IgG control antibody (IgG) (DIA‐AN, Cat. no. Q6007‐10 mg). For adoptive cell transfer, 1 × 10^7^ DC‐pulsed CD8^+^ T cells (i.e., tumor‐specific CD8^+^ T cells) were first suspended in 100 µL of PBS and then injected into the tumor‐bearing mice via the tail vein every 3 days for up to 12 days. PBS was used as a control. In addition, the tumor‐bearing mice were also treated with 5 mg kg^−1^ aPD‐L1 or IgG via intraperitoneal (IP) injection every 3 days for up to 12 days. At the end of the treatment, the mice were sacrificed by cervical dislocation, according to institutional guidelines, while the tumors were harvested, fixed, and paraffin‐embedded for further analysis.

### Drugs and Treatment

The γ‐secretase inhibitor DAPT (N‐[N‐(3,5‐difluorophenacetly L‐alanyl)]‐S‐phenylglycinet‐butyl ester, GSI‐IX) (Selleck, Cat. no. S2215‐30 mg‐lsx) was diluted in DMSO (4%) (Sigma, Cat. no. D2650‐100ML) and corn oil (96%) (Selleck, S6701‐100 mL). Anti‐PD‐L1 monoclonal antibody (aPD‐L1) (BioXCell, Cat. no. BP0101‐25MG) or anti‐IgG control antibody (aIgG) (DIA‐AN, Cat. no. Q6007‐10 mg) was diluted in PBS. In vivo jet/PEI regent (Polyplus‐transfection, Cat. no. 201–50G) was applied as a carrier for delivering YY1‐ or ICAM1‐targeting siRNA to liver tissues. A total of 40 µg of siRNA was suspended in 2′OMe in 100 µL of 5% glucose, while 6.4 µL of in vivo jet/PEI was diluted in 100 µL of 5% glucose. Finally, the diluted in vivo jet/PEI reagent was mixed with the siRNA or plasmid mixture and incubated at room temperature for 15 min. Thus, the complex was established according to the manufacturer's protocol with a final N/P ratio of 8.

### Generation of an Orthotopic HCC Tumor Model

Four‐week‐old male C57BL/6 mice were selected to construct orthotopic HCC models. First, 5 × 10^5^ WT/Hepa1‐6^N1ICD^/Hepa1‐6^shN1ICD^/Hepa1‐6^scr^ cells were resuspended in 15 µL of Matrigel in 10 µL of PBS, which was then injected into the left lobe of the liver.

For the PEI‐siRNA complex treatment experiment, the tumor‐bearing mice were randomly divided after 7 days post‐injection and were given either 2 mg kg^−1^ PEI‐siRNA or PEI‐plasmid via tail vein injection every 3 days up to three times or 10 mg kg^−1^ d^−1^ Notch1 inhibitor DAPT/DMSO solvent via subcutaneous injection, together with 5 mg kg^−1^ aPD‐L1 or IgG via intraperitoneal injection every 3 days for up to 3 times. The tumor size was monitored via animal ultrasound imaging (Vevo 2100, FUJIFILM VisualSonics) every 3 days for up to 3 times according to the manufacturer's instructions. After treatment, the mice were sacrificed, and their livers were harvested, weighed, fixed, and then embedded in paraffin for further immunohistochemistry studies.

### Adverse Gastrointestinal Side Effects

In the orthotopic HCC tumor mouse model, adverse gastrointestinal side effects included diarrhea and weight loss. Diarrhea manifests as a change in fecal characteristics and can be classified as normal, soft, mucinous, or watery. The degree of weight loss was graded by the percentage of decrease, which was defined as 0, 1–5%, 5–10%, 10–15%, or >15%.

### Statistical Analysis

The statistical analysis used in this study was completed by using GraphPad Prism version 10. Continuous variables between different groups were compared via Student's test or one‐way analysis of variance (ANOVA), and categorical variables were compared via the chi‐square test. The Kaplan‒Meier method and the log‐rank test were used to evaluate differences in progression‐free survival (PFS) and overall survival (OS) between groups. All the experiments were repeated at least 3 times. All values are expressed as the mean ±  standard error of the mean (SEM). A two‐tailed *p*‐value < 0.05 was considered statistically significant.

## Conflict of Interest

The authors declare no conflict of interest.

## Author Contributions

K.Z., F‐P.Z., C.Q., and Z‐X.S. contributed equally to this work. K.Z. and F‐P.Z. carried out the majority of the experiments and contributed equally to the paper; C.Q., Z‐X.S., and S.‐S.L. performed the animal experiments; F.‐P.Z., C.‐N.Y., and C.‐Q.L. carried out the RNA sequencing analysis and immunohistochemistry analysis; X.‐H.Y. and W.‐R.W. provided the clinical samples; C.L. and L.‐B.X. conceived the study, supervised the research, and wrote the manuscript. All the authors had access to the study data and reviewed and approved the final manuscript.

## Supporting information



Supporting Information

## Data Availability

The data are available from the corresponding author upon reasonable request. All data relevant to the study are included in the article or uploaded as supplementary information.
