# Supplementary material for: Targeting the Notch1‐YY1‐ICAM1 Signaling Axis Enhances the Efficacy of Immunotherapy in HCC by Activating CD8+ T‐Cell‐Driven Cancer Cell Pyroptosis
Source: Adv Sci (Weinh). 2025 Nov 21;13(7):e12845. doi: 10.1002/advs.202512845 (PMC12866794; doi:10.1002/advs.202512845)
Supplement: Supplementary file 1 — Supporting Information [file ADVS-13-e12845-s001.docx]

**Targeting Notch1-YY1-ICAM1 signaling axis enhances immunotherapy efficacy in HCC by activating CD8^+^ T cell driven cancer cell pyroptosis**

**SUPPLEMENTARY FIGURES**

**
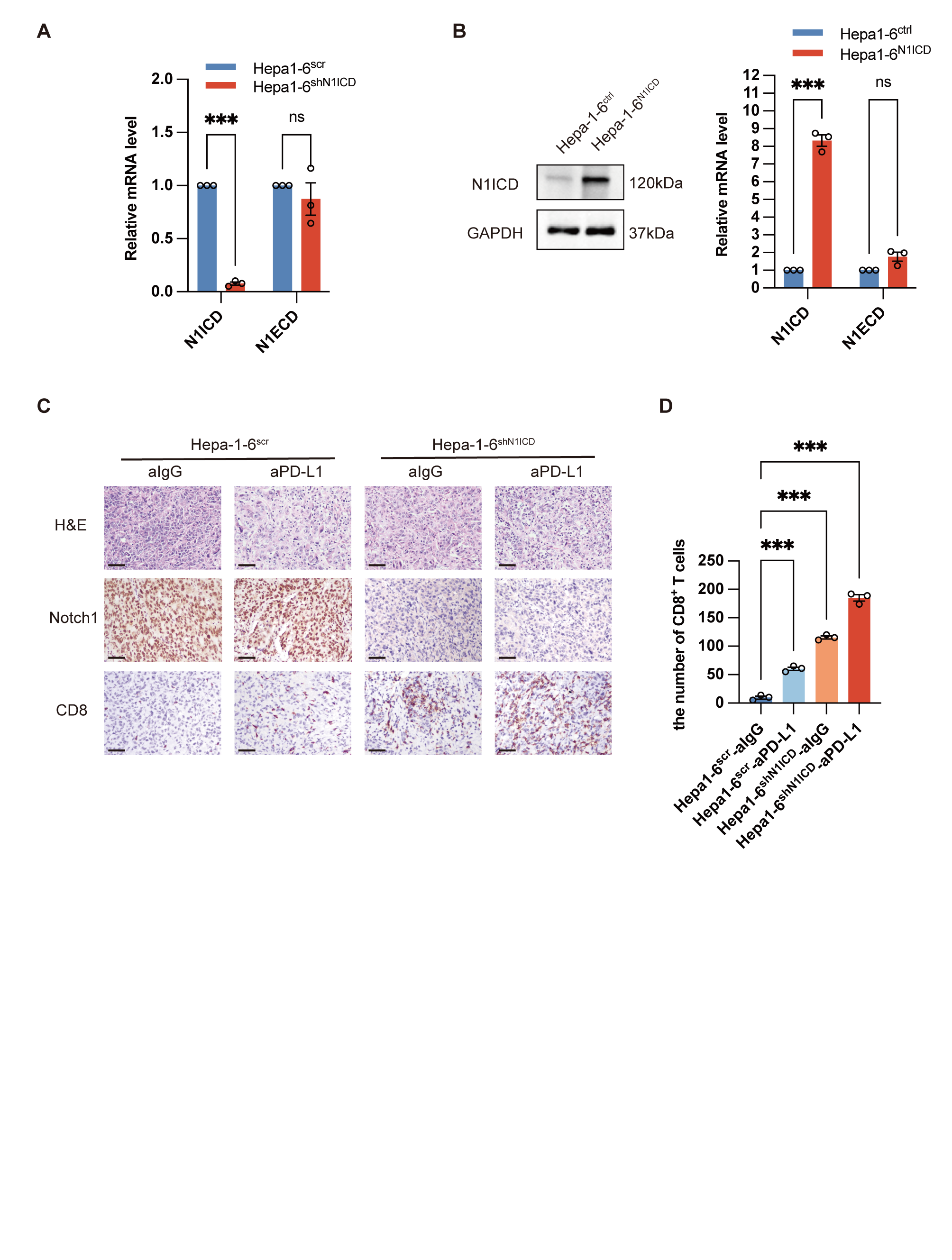
**

**Figure. S1 Expression of Notch1 predicts the immunotherapeutic response of anti-PD-1/PD-L1 in HCC patients.** (**A**) RT-qPCR confirmed the knockdown of N1ICD in Hepa-1-6 cells. (**B**) Both Western blot and RT-qPCR confirmed the overexpression of N1ICD in Hepa-1-6 cells. (**C**) Representative images of H&E-stained sections and immunohistochemical staining of CD8 and N1ICD from each group are given. (**D**) Bar chart shows the number of CD8^+^ T cells in each group. Means ± SEM are given. ns, non-significant difference. ***p < 0.001. (**A, B, D**) Student’s t test. Scale bar in (**C**) represents 100 μm.

**
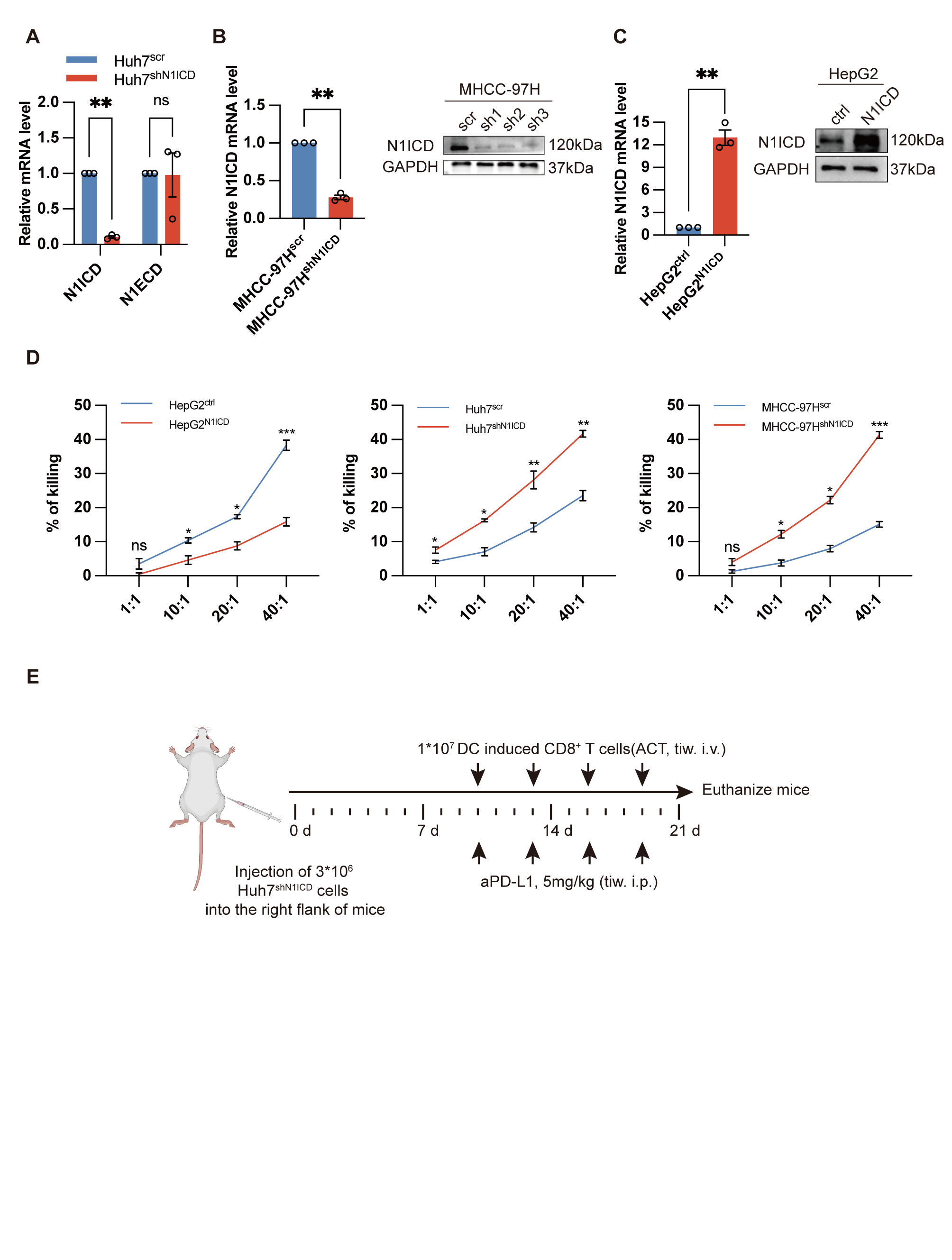
**

**Figure. S2 Schematic diagrams represent the preparation procedure of human tumor specific CD8^+^ T cells and the treatment plan of adoptive cell transfer therapy in HCC subcutaneous model.** (**A-C**) Both Western blot and RT-PCR confirmed the knockdown of N1ICD in Huh7/MHCC-97H cells and the overexpression of N1ICD in HepG2 cells. (**D**) LDH release assay of HepG2^N1ICD^/HepG2^ctrl^ cells or Huh7^shN1ICD^/MHCC-97H^shN1ICD^ cells/ Huh7^scr^/MHCC-97H^scr^ after co-culture with non-DC primed CD8^+^ T cells at different E/T ratios as indicated (n= 3 independent experiments). (**E**) The treatment strategy of adoptive cell transfer (ACT) therapy (i.e. tumor specific CD8^+^ T cells) with PD-1/PD-L1 inhibitor in HCC subcutaneous models. Means ± SEM are given. ns, non-significant difference. *p < 0.05, **p < 0.01, ***p < 0.001, ****p < 0.0001. (**A-D**) Student’s t test.

**
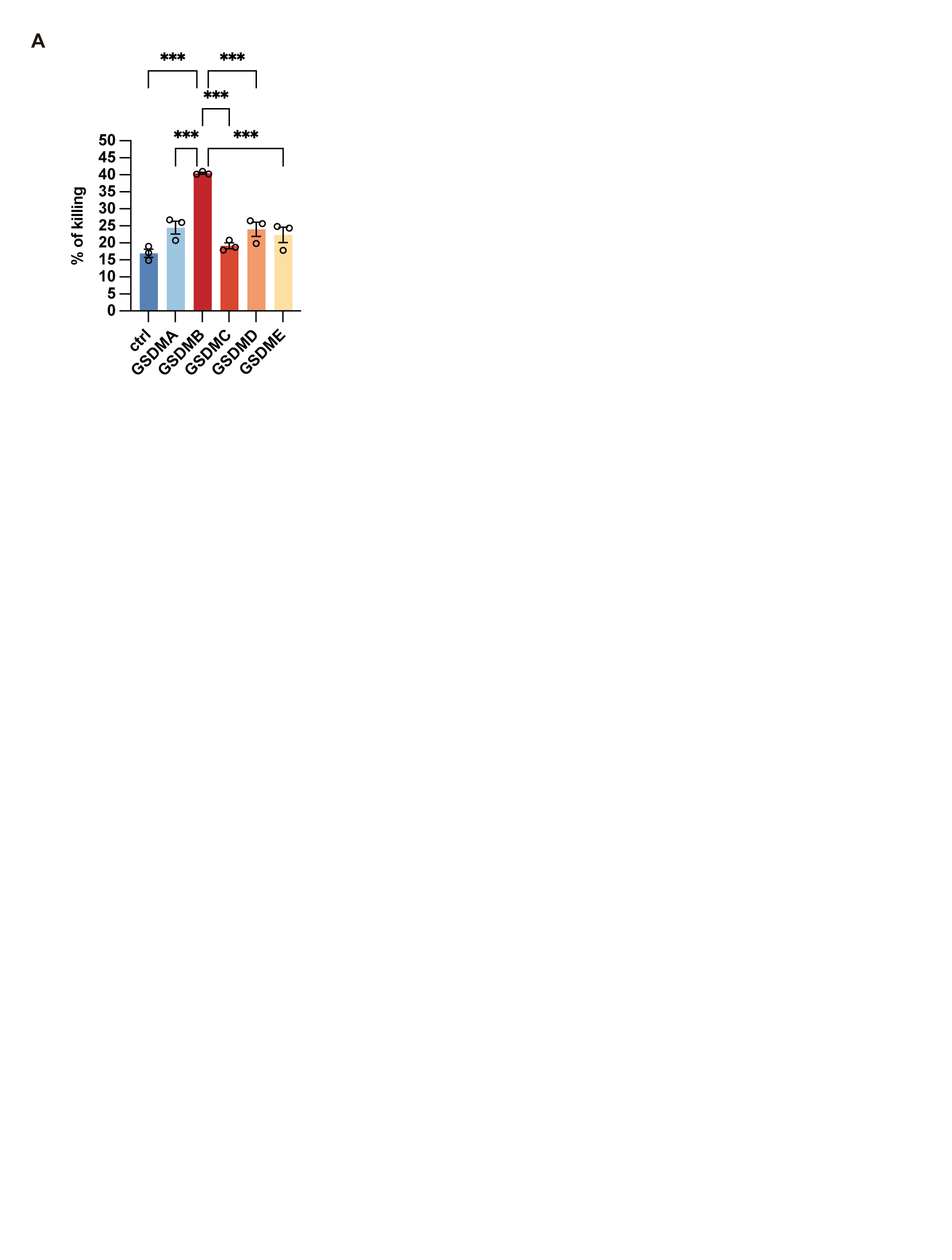
**

**Figure. S3 GSDMB is identified as a key executor of pyroptosis in the co-culture model.** (**A**) LDH release assay of HepG2^GSDMA/B/C/D/E^/HepG2^ctrl^ cells after co-culture with tumor specific CD8^+^ T cells (n= 3 independent experiments). Means ± SEM are given. ***p < 0.001. (**A**) Student’s t test.

**
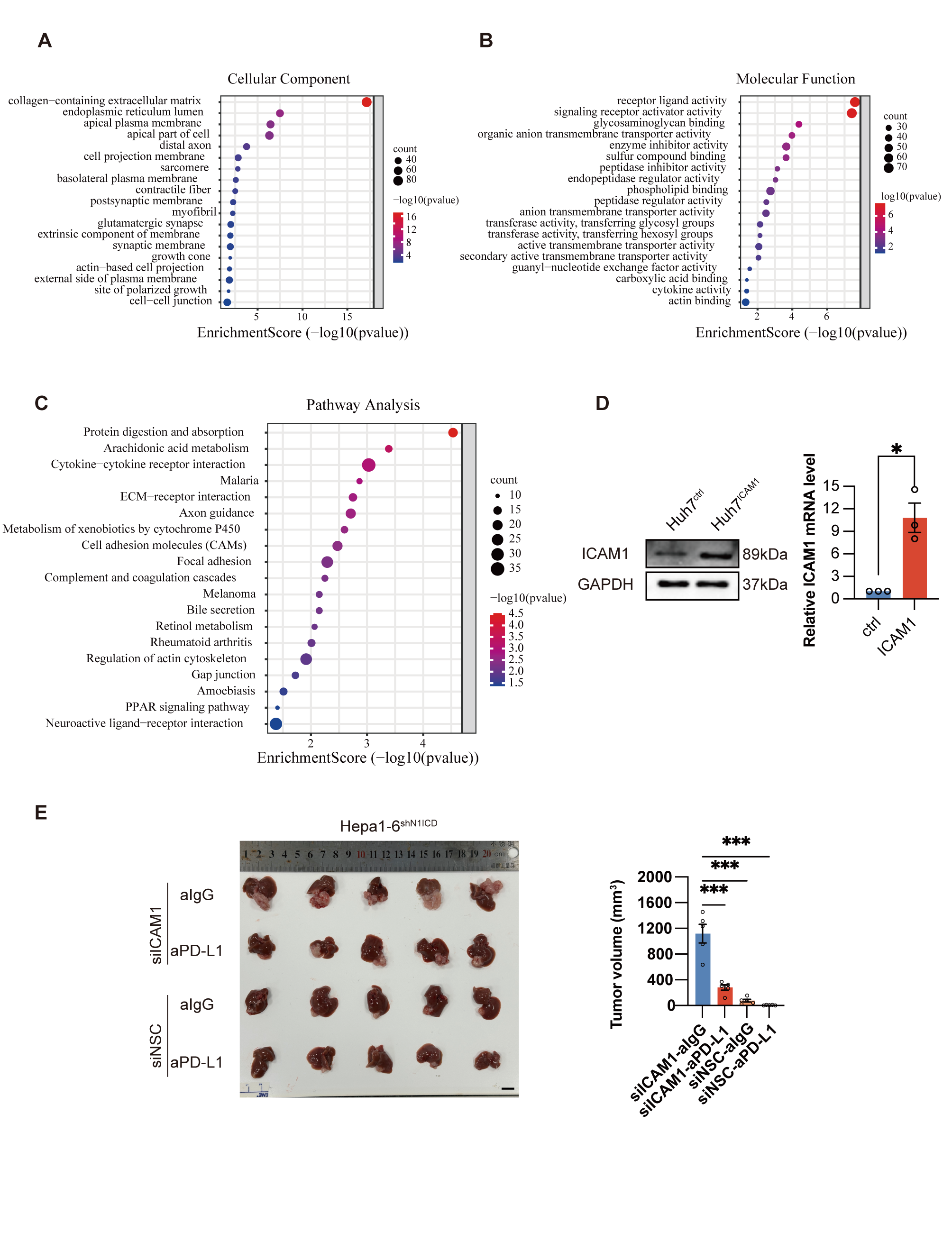
**

**Figure. S4 RNA-seq analysis indicates that the expression of ICAM1 is determined by N1ICD expression in HCC cells.** (**A-B**) GO enrichment analysis reveals a significant difference in some cellular component and molecular functions between Huh7^shN1ICD^ cells and Huh7^scr^ cells. (**C**) KEGG enrichment analysis revealed a significant enrichment in pathways that involved in protein digestion and absorption as well as cell adhesion molecule etc, in Huh7^shN1ICD^ cells. (**D**) Both Western blot and RT-PCR confirmed the overexpression of ICAM1 in Huh7 cells. (**E**) Representative gross tumor image from each treatment group is given. Bar chart represents the final tumor volume in each group (n=5 mice per group). *p < 0.05. (**A-E**) Student’s t test. Scale bar in (**E**) represents 1 cm.

**
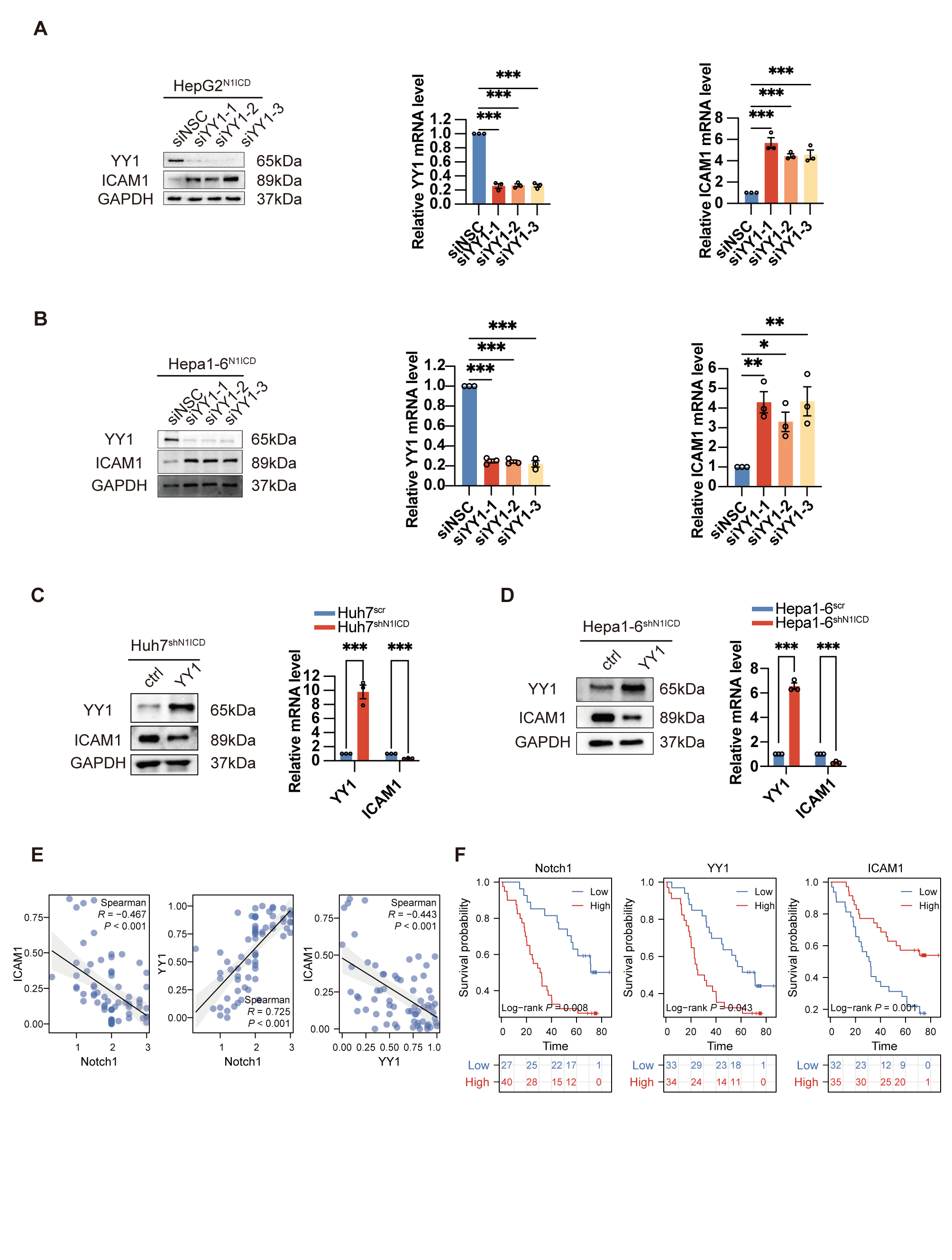
**

**Figure. S5 NI1CD transcriptionally up-regulates transcriptional repressor YY1 expression to repress *ICAM1* gene transcription.** (**A-B**) Western blot and RT-qPCR analysis of the YY1 and ICAM1 expression in HepG2^N1ICD^/Hepa1-6^N1ICD^ cells transiently transfected with YY1-targeting siRNA or non-silencing control siRNA. (**C-D**) Western blot and RT-qPCR analysis of the YY1 and ICAM1 expression in Huh7^shN1ICD^/Hepa1-6^shN1ICD^ cells transiently transfected with either empty control vector or YY1 overexpression vector. (**E**) The expression of ICAM1 and Notch1 was negatively correlated (left), the expression of YY1 and Notch1 was positively correlated (middle)，the expression of YY1 and ICAM1 was negatively correlated in our HCC cohort 2 (n= 67 patients) (right). (**F**) High expression of Notch1 (left) and YY1 (middle) correlated with poor overall survival, and low expression of ICAM1 (right) correlated with poor overall survival in our HCC cohort 2 (n=67 patients). Means ± SEM are given. *p < 0.05, **p < 0.01, ***p < 0.001. (**A-D**) Student’s t test. (**E**) Spearman correlation study. (**F**) Log-rank test.


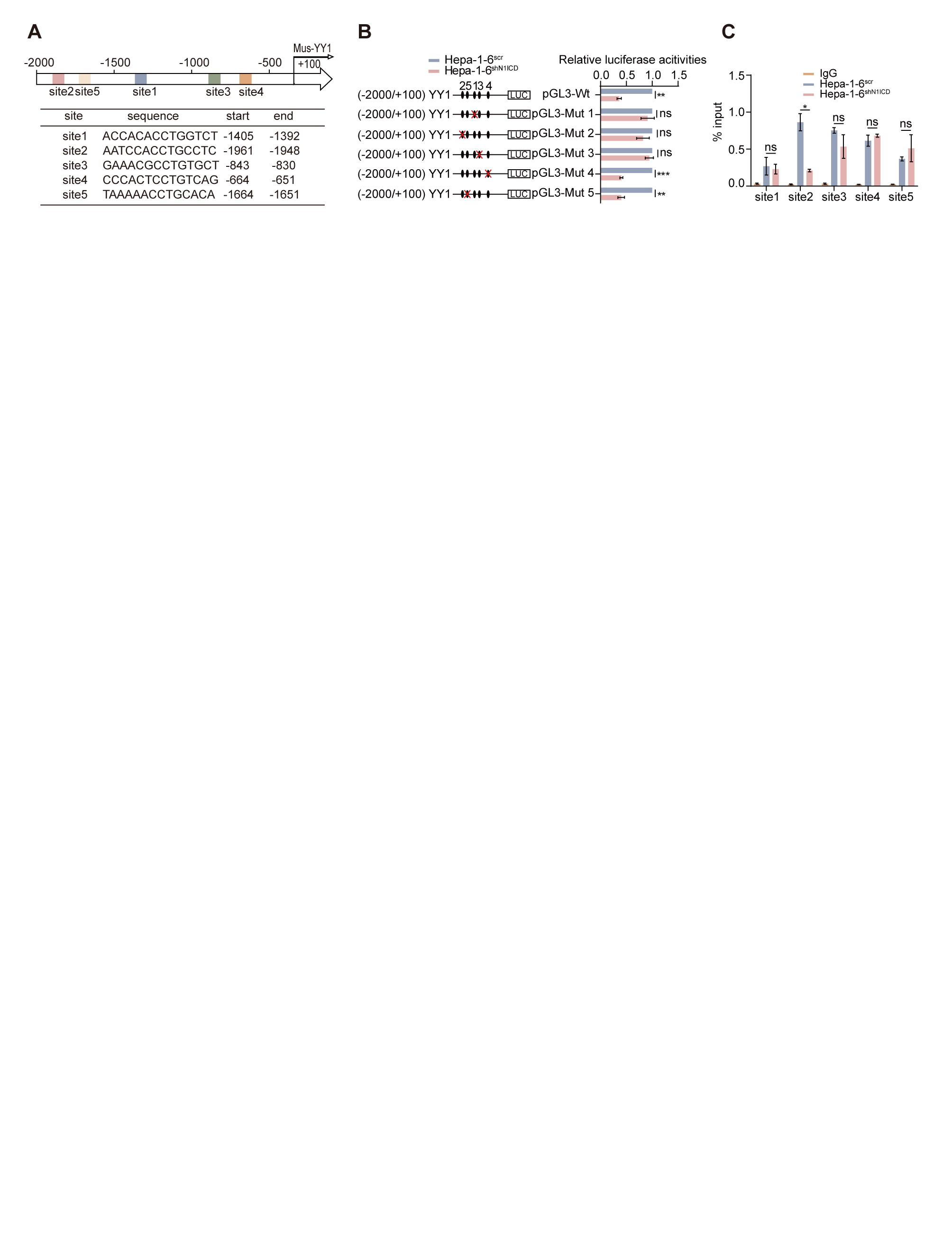


**Figure. S6. N1ICD directly binds to the YY1 promoter to regulate its transcription in mouse HCC cells.** (**A**) Schematic diagram represents the putative NI1CD binding sites in mouse YY1 promoter. (**B**) Luciferase assay of Hepa1-6^shN1ICD^ cells transfected with a luciferase reporter vector containing either a full-length mouse YY1 promoter or mutated YY1 promoter sequence. (**C**) ChIP assays indicated that the N1ICD binding to its 2^nd^ putative site was decreased in Hepa1-6^shN1ICD^ cells as compared to scramble transfected cells. non-significant difference, ns, non-significant difference. *p < 0.05, **p < 0.01, ***p < 0.001. (**B-C**) Student’s t test.

**
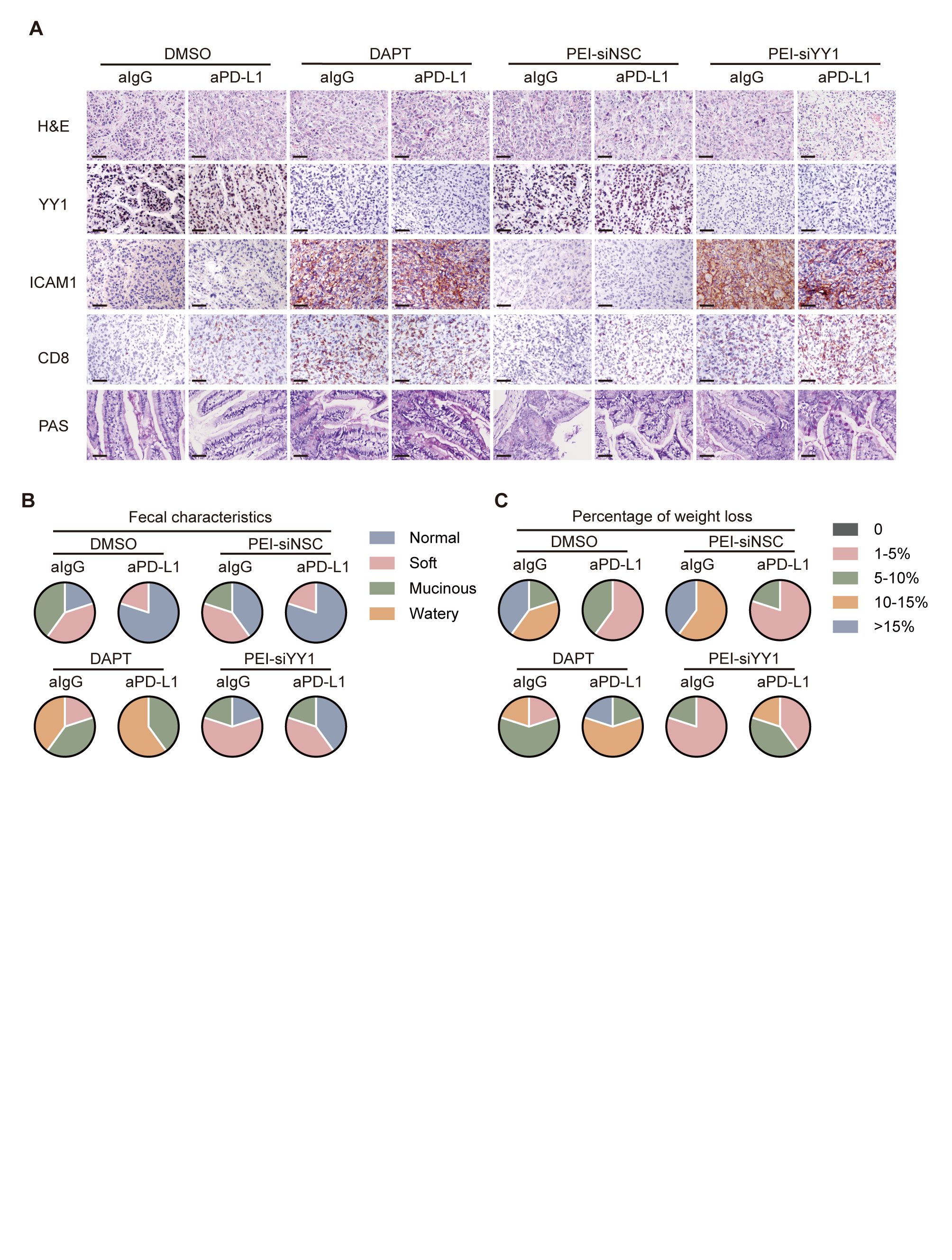
**

**Figure. S7.** **Combined treatment of Notch1 inhibitor and PD-L1 antibody cause significant toxic side effects on HCC orthotopic tumor bearing mice.** (**A**) Representative H&E-stained sections, PAS-stained sections and immunohistochemistry staining of YY1, ICAM1 and CD8 are given. (**B-C**) Pie charts indicate the fecal characteristics（**B**）and the percentage of weight loss（**C**）in the tumor bearing mice after treated with different drug combination as indicated in figure 7A. Means ± SEM are given. Scale bars in (**A**) 100 μm.

**Table. S1** Table shows the clinicopathological details of HCC patients who received adjuvant anti-PD-1/PD-L1 antibody treatment with high or low Notch1 expression in our cohort.

**Table. S2** The siRNA target sequences of YY1 gene.

**Table. S3** The sequences of primers for quantitative real-time PCR

**Table. S4** The list of antibodies used in current study.

**Table. S5** The genes predicted in Fig 6A.

**Table. S6** The sequences for the promotor of YY1 luciferase reporter plasmids construction.

**Table. S7** The sequences for the promotor of ICAM1 luciferase reporter plasmids construction.

**Table. S8** The sequences of primers for the predicted binding sites in YY1 promotor.

**Table. S9** The sequences of primers for the predicted binding sites in ICAM1 promotor.

**SUPPLEMENTARY TABLES**

Supplementary Table 1. The clinicopathological details of HCC patients who received adjuvant PD-1/PD-L1 antibody treatment with high or low Notch1 expression in our cohort

| Variables | All cases | Notch1 expression | |
| --- | --- | --- | --- |
|  |  | Low | High |
| Gender |  |  |  |
| Male | 30 | 12 | 18 |
| Female | 4 | 1 | 3 |
| Age (years) |  |  |  |
| <60 | 24 | 9 | 15 |
| ≥60 | 10 | 4 | 6 |
| HBV |  |  |  |
| Negative | 2 | 0 | 2 |
| Positive | 32 | 13 | 19 |
| Cirrhosis |  |  |  |
| No | 13 | 5 | 8 |
| Yes | 21 | 8 | 13 |
| AFP (ng/ml) |  |  |  |
| <400 | 12 | 3 | 9 |
| ≥400 | 22 | 10 | 12 |
| TNM stage |  |  |  |
| I-II | 21 | 8 | 13 |
| III-IV | 13 | 5 | 8 |
| Response |  |  |  |
| CR | 1 | 1 | 0 |
| PR | 3 | 1 | 2 |
| SD | 12 | 9 | 3 |
| PD | 18 | 2 | 16 |

Supplementary Table 2. siRNA target sequences of YY1 gene

| siRNA | Target sequences (5’-3’) |
| --- | --- |
| Mus-siYY1-1 | CGGCGACGACGACUACAUA |
| Mus-siYY1-2 | GAUGAUGCUCCAAGAACAA |
| Mus-siYY1-3 | CGUUGAGAGCUCAAAGCUA |
| Hsa-siYY1-1 | CGACGACTACATTGAACAA |
| Hsa-siYY1-2 | GATGATGCTCCAAGAACAA |
| Hsa-siYY1-3 | AGAAGCAGGTGCAGATCAA |

Supplementary Table 3. Sequences of primers for quantitative real-time PCR

| Gene | Forward primer (5’-3’) | Reverse primer (5’-3’) |
| --- | --- | --- |
| Hsa-N1ICD | ACCAATACAACCCTCTGCGG | GGCCCTGGTAGCTCATCATC |
| Hsa-YY1 | AAAGAAACTTCCTCCTGGAG | GGCTTCATTCTAGCAAATTCTG |
| Hsa-ICAM1 | TGACCGTGAATGTGCTCTCC | TCCCTTTTTGGGCCTGTTGT |
| Hsa-VDR  Hsa-GSDMB | GGAAGGCACTATTCACCTG  GCTGAAGAGGGAACTACCCTT | CATCTGTCAGAATGAACTCCT  TCCTTTACCGTCTCCAGAGTTT |
| Hsa-IL18R1 | GGTATTACTCCTGCGTGCA | GAACTATATTACTGCGATCTTCCAC |
| Hsa-CD47 | GTATTGCGGCGTGTATACC | GTCCAAGTAATTGTGCTAGAGC |
| Hsa-LGALS3 | TCTTCTGGACAGCCAAGTG | TTATAAGGCACAATCAGTGGC |
| Hsa-ANXA1 | GCAGGAATATGTTCAAACTGTG | GATGGATTGAAGGTAGGATAGG |
| Hsa-TNFSF4 | ACCTCATGGTATCACATCGG | TGAGGATGAAACCTTTCTCCT |
| Hsa-ZP3 | TAACAAAGGTGACTGTGGC | TTCTTCTGTCACATGCCTG |
| Hsa-IL7R | GACCTAACCACTATAGTTAAACCTG | CACCACAAAGTCATTGGCTC |
| Hsa-CD81 | GTCAACAAGGACCAGATCG | TCTCGTGGAAGGTCTTCAC |
| Hsa-FUT7 | GATGAATAATGCTGGGCACG | ACAAGGATGGTGATCGTGG |
| Hsa-MYB | AATGTCTCCAGTCATGTTCC | ATAGTGTCTCTGAATGGCTG |
| Hsa-IL18 | TGACCAAGTTCTCTTCATTGAC | GGTGCATTATCTCTACAGTCAG |
| Hsa-PRKCZ | CTTTAACAGGAGAGCGTACTG | CAGTTGATGCACCTGTAGC |
| Hsa-FCER1G | GAATTGTCCTCACCCTCCT | CATCTGATTTCTCATAGCTGGT |
| Hsa-HLA-DMB | TCTTGGTGTGATCAGCTGG | GGAAATGTGCCATCCTTCTG |
| Hsa-F2RL1 | CCTTTGGACTCGATCTTGG | CTCCTCCATATCCAACACCC |
| Has-GAPDH | GGAGCGAGATCCCTCCAAAAT | GGCTGTTGTCATACTTCTCATGG |
| Mus-N1ICD | TGGATGACCTAGGCAAGTC | TTCTGCATGTCCTTGTTGG |
| Mus-YY1 | CCCTTTCAGTGCACATTCG | GGTATGGATTCGCACATGTG |
| Mus-ICAM1 | TCCGCTACCATCACCGTGTAT | TAGCCAGCACCGTGAATGTG |
| Mus-GAPDH | AGGTCGGTGTGAACGGATTTG | TGTAGACCATGTAGTTGAGGTCA |
| Mus-N1ECD | ACGTAGTCCCACCTGCCTAT | CAGGTGCCCTGATTGTAGCA |

Supplementary Table 4. Antibodies used in current study

| Target | Company | Application | Catalog No. |
| --- | --- | --- | --- |
| Notch1 | Cell Signaling Technology | WB | 3608s |
| Notch1 | Abcam | IHC | ab8925 |
| ICAM1 | Abcam | WB, IHC(Hsa) | ab282575 |
| ICAM1 | Abcam | WB, IHC(Mus) | ab222736 |
| ICAM1 | eBioscience | Neutralization | 14-0549-82 |
| YY1 | Abcam | WB, IHC | ab109237 |
| YY1 | Cell Signaling Technology | ChIP | 46395S |
| GAPDH | Cell Signaling Technology | WB | 2118S |
| H3 | Cell Signaling Technology | ChIP | 4620S |
| IgG | Cell Signaling Technology | ChIP | 3900S |
| CD8 | ZSGB-BIO | IHC | ZA-0508-0.2 |
| BV650-labeled CD8 | BD Biosciences | FACS | 563822 |
| PE-labeled CD107a | BD Biosciences | FACS | 560948 |
| PD-L1 | Cell Signaling Technology | IHC | 13684S |
| Dako REAL EnVision Detection System | DAKO | IHC | K5007 |
| InVivoPlus anti-mouse PD-L1 (B7-H1) | BioXcell | Blocking | BP0101 |

Supplementary Table 5. The genes predicted in Fig 6A.

| Databases | Genes |
| --- | --- |
| Cistrome DB | RelA MEF2A ESR1 MED1 MAX RXRG TCF12 EP300 CBFB MBD1_isoform2 NFKB1 SPI1 PML ZFX JMJD1C MXI1 POLR2A ZNF24 PBX3 CBX5 PKNOX1 T HMGN3 RXRA BRD4 TCF7L1 CTBP2 ZNF639 RUNX1 FUS EGR1 TTF1 MYC LMO2 NIPBL SIN3A HCFC1 CBX3 BCL6 Pax-5 VDR CEBPB TERC ZBTB16 RARA MYB TFAP2A E2F5 STAT5B STAT4 SSU72 FLI1 NFIA ZNF766 EBF1 ZBTB11 NR2F2 UBTF BHLHE40 TFAP2C KDM1A ICE1 TCF4 JMJD6 NOTCH1 AFF4 JUND STAT1 NFKB2 SMARCC1 SUMO2 IKZF1 IRF1 YY1 TBL1XR1 ZBTB7A RCOR1 ZNF22 ZNF143 TAL1 IRF4 CTCF REST GRHL2 FOXA1 SMARCA4 TCF7L2 |
| PROMO | GR-beta TFII-I C/EBPbeta LEF-1 ENKTF-1 STAT4 c-Ets-1 RXR-alpha p53 T3R-beta1 GR-alpha AP-2alphaA GATA-2 VDR GATA-1 NF-1 FOXP3 E2F-1 NFI/CTF POU2F1 HNF-4alpha Pax-5 MYB IRF-2 PXR-1:RXR-alpha YY1 TFIID HNF-3alpha GR XBP-1 GCF AR RAR-beta:RXR-alpha PR B PR A C/EBPalpha RAR-beta Sp1 c-Ets-2 NF-AT2 NF-kappaB RelA STAT1 RBP-Jkappa Ik-1 EBF NF-kappaB1 MEF-2A IRF-1 NF-AT1 ER-alpha ETF Elk-1 c-Jun MAZ PPAR-alpha:RXR-alpha AhR WT1 ATF3 PEA3 HNF-1C HOXD9 HOXD10 HNF-1B TBP AhR:Arnt USF2 NF-AT1 SRY TCF-4E ATF-1 STAT5A RAR-alpha1 AP-1 c-Fos E2F GATA-3 |
| hTF-target (liver) | CEBPA CEBPB CREB1 CTCF ELF1 FOXA2 GABPA HDAC2 HNF4A HNF4G JUN MAX MAZ MXI1 NFE2 NR2C2 NR2F2 POLR2A RAD21 RXRA SIN3A SMC3 SP1 SUZ12 TAF1 TAL1 TBP USF1 USF2 VDR YY1 ZBTB7A ZEB1 |

Supplementary Table 6. The sequences for the promotor of YY1 luciferase reporter plasmids construction

| Binding site | Wild sequences | Mutant sequences |
| --- | --- | --- |
| Has-site1 | ACCGGGAAGCGGGAGGCGGTG | ACCGGGAAataaagaataaTG |
| Has-site2 | GGTGCCCGGGGCCGCGCGGAC | GGTGCCCGaaattatataaAC |
| Has-site3 | GGGAGGGAGGGGCCGGCCGGGA  GCGGGGTTGAGGCGGGT | GGGAGGGAaaaattaattaGGA  ataaGGTcagaataaGT |
| Mus-site1 | ACCACACCTGGTCT | ACCACgatcaaTCT |
| Mus-site2 | AATCCACCTGCCTC | AATCCgtagaaCTC |
| Mus-site3 | GAAACGCCTGTGCT | GAAACattcagGCT |
| Mus-site4 | CCCACTCCTGTCAG | CCCACctactgCAG |
| Mus-site5 | TAAAAACCTGCACA | TAAAAgaagatACA |

Supplementary Table 7. The sequences for the promotor of ICAM1 luciferase reporter plasmids construction

| Binding site | Wild sequences | Mutant sequences |
| --- | --- | --- |
| Has-site1 | TGGATGGCC | TGagcaatC |
| Has-site2 | CTCCATGGCG | CTCtgcaatG |
| Mus-site1 | AGCTGCCATGA | AGactatgTGA |
| Mus-site2 | GGATGGCGGTC | GGccattaGTC |

Supplementary Table 8. The sequences of primers for the predicted binding sites in YY1 promotor

| Sites | Forward primers (5’-3’) | Reverse primers (5’-3’) |
| --- | --- | --- |
| Hsa-1 | TTGTGGCTGTTGCACCGCGAA | CGATTCTCCTCTCGGCCAAT |
| Hsa-2 | TCCCTCCCTTCTCCTCAGGC | CTACAGCCGCTGCCTTGGGA |
| Has-3 | GGAAGCACAGGCGATTCT | CTACAGCCGCTGCCTTGGGA |
| Mus-1 | CAAATAAGATACACTTGGCTC | GTGTTTCTTCTGTGCTTCC |
| Mus-2 | TCACTCTGTAGACCAGGCTG | CGCGTTTAATCCCAGCACTT |
| Mus-3 | AAAGAGCCCGTGGCAGCA | GCAATAAAGTCTGCTCTGAC |
| Mus-4 | TGGGTCGCGCGGAAAGTT | GCGGTGCAACAGTGACAA |
| Mus-5 | CTATACACGTAGCCAAGGCT | CCAAACTTAAACAGTTGGGC |

Supplementary Table 9. The sequences of primers for the predicted binding sites in ICAM1 promotor

| Sites | Forward primers (5’-3’) | Reverse primers (5’-3’) |
| --- | --- | --- |
| Hsa-1 | GAATTCAGAACTCCTCAGCC | AGAAGGGGAAGCGAAGGGTC |
| Hsa-2 | GACCCTTCGCTTCCCCTTCT | GCCTAAGGCTTTCCTGTTGT |
| Mus-1 | TCAGATCGCTAGAGCTGGAG | GCAGAGAACACAGGTTCAATT |
| Mus-2 | CAGGACTTGATTTCGGATCC | TAGTCCCCTGCGACATCA |
